# Povidone iodine suppresses LPS-induced inflammation by inhibiting TLR4/MyD88 formation in airway epithelial cells

**DOI:** 10.1038/s41598-022-07803-2

**Published:** 2022-03-07

**Authors:** Seung Hoon Lee, Mi-Ra Choi, Jaein Chung, Seung-Hyeon Choi, Soo Kyoung Park, Yong Min Kim

**Affiliations:** 1 Department of Otorhinolaryngology-Head and Neck Surgery, Research Institute for Medical Science, Chungnam National University School of Medicine, 282 Munhwa-ro, Jung-gu, Daejeon, 35015 South Korea; 2 Department of Medical Science, Chungnam National University School of Medicine, Daejeon, South Korea; 3 Department of Otorhinolaryngology-Head and Neck Surgery, Chungnam National University Hospital, Daejeon, South Korea

**Keywords:** Cell biology, Chemical biology, Drug discovery, Immunology, Diseases, Health care, Molecular medicine

## Abstract

Povidone-iodine (PVP-I) is an antiseptic and a disinfectant with broad-spectrum antimicrobial activity against various pathogens. However, it is unclear whether PVP-I nasal instillation can suppress mucosal inflammation in non-eosinophilic chronic rhinosinusitis (CRS) mice. This study aimed to explore the anti-inflammatory effects and underlying molecular mechanism of PVP-I on lipopolysaccharide-stimulated airway epithelial cells and investigate whether nasal instillation of PVP-I can suppress mucosal inflammation in non-eosinophilic CRS mice. Inflammation-related molecules in the nasal epithelial cells and non-eosinophilic CRS mice were measured by enzyme-linked immunosorbent assay, western blotting, quantitative real-time polymerase chain reaction, immunoprecipitation, and histopathological analysis. PVP-I blocked expressions of various inflammation-related molecules, such as NLRP3, NF-κB-p65, caspase-1, and IL-1β. Translocation of NF-κB to the nucleus, and assembly of NLRP3/ASC complexes in the nasal epithelial cells and non-eosinophilic CRS mice were also restricted. Notably, PVP-I strongly blocked the receptor co-localization of TLR4 and MyD88 in the epithelial cells of nasal mucosa. We demonstrated that PVP-I significantly attenuated inflammatory molecules and cytokines via blocking the formation of TLR4 and MyD88 complexes during LPS-induced mucosal inflammation in non-eosinophilic CRS.

## Introduction

Chronic rhinosinusitis (CRS) is a persistent inflammatory disease of the nasal and paranasal sinuses mucosa^1^. CRS is a heterogeneous disorder with distinct pathophysiologic mechanisms, and evidence from several studies has revealed different endotypes^2–4^. The selective expression of type 1, 2, or 3 immune responses is associated with increasing CRS heterogeneity^5–8^. An endotype of CRS with prominent type 2 immune responses is associated with eosinophilic infiltration into sinonasal mucosa and can benefit from treatment with systemic steroids and biologics, whereas non-eosinophilic CRS shows resistance to these therapy^9–12^.

Nucleotide-binding oligomerization domain-like receptor family pyrin domain-containing 3 (NLRP3) could combine with the Apoptosis-associated speck-like protein containing a caspase recruitment domain (ASC) and the pro-caspase-1 to compose the NLRP3 inflammasomes^13^. NLRP3 inflammasomes play essential roles in the innate and adaptive immune responses serving as a pattern-recognition receptor and a trigger for caspase-1 activation and the maturation of IL-1β and IL-18^14,15^.

The NLRP3 and its downstream IL-1β were identified as potent inducers of neutrophilic inflammation in various inflammatory diseases^16,17^. Previous studies showed that NLRP3 was highly expressed in nasal polyps (NPs) from subjects with CRSwNP, and significantly correlated with neutrophilic nasal polyps^18,19^. Wei et al. reported that macrophages and epithelial cells were the primary sources of NLRP3 in the polyp tissues^19^.

Povidone-iodine (PVP-I) is an antiseptic and a disinfectant with broad-spectrum antimicrobial activity. It has been used as a topical treatment and surgical scrubs for several decades^20–22^. PVP-I consists of a complex of povidone, hydrogen iodide, and elemental iodine^23^.

PVP-I is effective in reducing edema or tissue inflammation and promoting wound healing, as well as eradication of most pathogens^24,25^. Here, PVP-I was more effective in non-eosinophilic CRS than its eosinophilic counterpart in terms of decreasing sinus discharge and improving olfaction and up to a 17% reduction in serum inflammatory markers was measured post-PVP-I rinsing^26^.

Although non-eosinophilic CRS has been associated with various bacterial infections, refractory CRS has been revealed to be closely associated with Gram-negative bacterial infection, and the endotoxins and LPS released by Gram-negative bacteria are considered as important pathogenic mechanisms^27,28^. While endotoxin may be a noninfectious inflammatory factor, it can regulate the release of inflammatory mediators, resulting in CRS^27,29^.

Here, we explore the anti-inflammatory effects and the underlying molecular mechanism of PVP-I on LPS-stimulated airway epithelial cells and investigate whether nasal instillation of PVP-I can suppress mucosal inflammation in non-eosinophilic CRS mice.

## Materials and methods

### Chemicals

Adenosine triphosphate (ATP, A2383), lipopolysaccharide (LPS; L2630; phenol extracted from Escherichia coli (O111:B4); serotype), and Poly (vinylpyrrolidone)–iodine complex (PVP-I) were purchased from Sigma (St. Louis, MO, USA).

### Cell cultures, human nasal airway epithelial primary cells

A549 cells (human lung airway epithelial) and RPMI 2650 cells (airway epithelial, human nasal septum) were cultured at 37 °C, and 5% CO_2_ in RPMI medium (Gibco, Gaithersburg, MD USA) supplemented with 10% FBS (Cat No:16000044; Lot No:2351219P; USA; Gibco), 100 U/mL penicillin, and 100 μg/mL streptomycin (Gibco). For preparation, primary human nasal epithelial cells (pHNECs, n = 19) were derived from patients undergoing elective endoscopic sinus surgery and were provided by the Department of Otorhinolaryngology-Head and Neck Surgery, Chungnam National University Hospital. The pHNECs were incubated overnight in 1% Dispase II (Roche, Belmont, CA, USA) at 4 °C, digested with 0.25% Trypsin–EDTA (Gibco) for 15 min at 37 °C, and neutralized with 10% FBS. The digested tissue was passed through a 70 µM cell strainer (SPL, Pocheon, Korea) to remove the undigested tissue for collecting pHNECs and washed twice with RPMI medium. Then, the tube was centrifuged at 1300 rpm for 5 min. The obtained pHNECs were cultured in airway epithelial cell growth media (PromoCell, Heidelberg, Germany) supplemented with a mixture of amphotericin B (25 μg/mL), penicillin G (10,000 U/mL), and streptomycin (10,000 μg/mL). The cells were placed in a humidified chamber and incubated at 37 °C, 5% CO_2_, and 95% air for 2 weeks. The second cell passage provided all of the cells used in this study. The cells were stimulated with LPS (1000 ng/mL) and ATP (5 mM) for 24 h, and PVP-I (0.1%) was additionally added to the cell culture for 1 h after LPS or ATP stimulation or both.

### Animal study

Animal experiments were conducted using 7-weeks-old male C57BL/6 mice with 18–20 g body weight (Orient Bio Inc., Seungnam, Republic of Korea). This study protocol was approved by the Chungnam National University's Institutional Animal Care and Use Committees (CNUH-020-A0033). The mice were maintained under specific-pathogen-free conditions, consisting of a 12-h light/dark cycle, a temperature of 18–23 °C, and humidity of 50–60%. Food and water were available ad libitum. All methods were carried out in accordance with relevant guidelines and regulation and are reported in accordance with ARRIVE guidelines. After 1 week of acclimation in an animal facility, mice were divided into four groups (*n* = *40*): control (*n* = *10*), LPS (*n* = *10*) group, LPS+ intraperitoneal (IP) of dexamethasone (DEX) at 1 mg/kg (*n* = *10*), and LPS + intranasal (IN) of PVP-I at 0.1% (*n* = *10*).

Based on power analysis, the minimum sample size was estimated at 5. Every group had more than 5 animals. Separate groups of animals were necessary for specific sub-analysis, thus we were able to collect some common tissues from all of them. Wherever possible, these samples were also included to increase confidence and robustness of our data, thus some analysis had larger sample sizes. LPS was dissolved in Dulbecco's phosphate-buffered saline (DPBS) and injected intranasally (10 µg/kg) in 20-μL DPBS with a 10‑μL pipette three times a week for three consecutive months. Then, PVP-I was dissolved in DPBS and administered intranasally once per day for 7 days, and DEX administered intraperitoneally three times per day for 7 days. The animals were sacrificed 7 days after sensitization with DEX and PVP-I treatment (see Fig. 6a for a timeline). After partial tracheal resection under deep anesthesia, a micropipette was inserted into the posterior choana through the tracheal opening in the upper airway direction. Each nasal lavage fluid (NLF) was gently perfused with 200 μL DPBS, and fluid from both nostrils was collected and centrifuged for IL-1β enzyme-linked immunosorbent assay (ELISA). The head of each mouse was removed and fixed in 4% paraformaldehyde. Serial 4 μM thick sections were cut on a paraffin microtome designed for histological analysis using H&E, IHC, and PAS. The nasal mucosa tissue was meticulously excised using a small curet and micro-forceps. The harvested nasal mucosa was immediately soaked in T-PER Tissue lysis buffer for immunoblot analysis.

### Cytotoxicity measures

A549 and RPMI2650 cells were plated as stated above in clear 96-well plates. After incubation time, the ATP, LPS, and PVP-I were treated in varying concentrations and incubated for another 24 h. After completion, the cell suspension was discarded, and 1 mg/mL MTT solution was added and incubated for 4 h, and then DMSO was added. The absorbance was then taken at 570 nM. The absorbance of each well was measured using a spectrophotometer (Versamax microplate reader, Molecular Device, Sunnyvale, CA, USA) at a wavelength of 570 nM, and expressed as a percentage of the control.

### Immunoblot

The protein samples from airway epithelial cells (A549 and RPMI2650 cells) were prepared in radio immunoprecipitation assay (RIPA) lysis buffer (5 mM sodium fluoride, 10 μg/mL phenylmethylsulfonylfluoride, and 1 mM sodium vanadate) containing a protease inhibitor cocktail (Roche, Mannheim, Germany). Mouse mucosa tissue protein samples were prepared in T-PER Tissue lysis buffer (78510, Thermo Fisher Scientific, T-PER Tissue lysis Protein Extraction Reagent). All samples were separated on 12% SDS–polyacrylamide gels (SDS-PAGE) and then transferred to PVDF membranes, blocked with 5% w/v nonfat dry milk in PBS containing 0.5% Tween-20. The primary antibodies used were actin (CS-8457; rabbit; monoclonal; 1:1000; Cell Signaling, Danvers, MA, USA), caspase-1 (MA5-32909; mouse; monoclonal; 1:1000; Thermo Fisher Scientific, Waltham, MA, USA), IL-1β (GTX74034; mouse; polyclonal; 1:1000; genetex, USA), IκB-α (CS-4814; mouse; monoclonal; 1:1000; Cell Signaling), lamin B1 (AB65986; rabbit; polyclonal; 1:1000; abcam, United Kingdom), NF-κB-p65 (8242; rabbit; monoclonal; 1:1000; Cell Signaling), NLRP3 (AG-20B-0014; mouse; monoclonal; 1:1000; Adipogen, SA, Switzerland), and tubulin (T8203; mouse; monoclonal; 1:1000; Sigma).

### Histopathology

For immunocytochemistry, Cells were fixed by incubation with 4% paraformaldehyde at room temperature for 30 min. Fixed cells were rinsed in PBS, treated with 0.5% bovine serum albumin for 30 min, and then incubated overnight at 4 °C with rabbit anti-NLRP3, ASC, NF-κB-p65, IL-1β, TLR4 (SC-10741, Santa Cruz Biotechnology, Santa Cruz, USA) and MyD88 (SC-74532, Santa Cruz Biotechnology, USA). antibodies (each diluted 1:200). They were then incubated for 2 h with Alexa 488 and 594 fluor-conjugated secondary antibody (dilution 1:500). The cells were then stained with 1 μM Hoechst staining solution (Invitrogen, CA, USA) for 10 min at RT and then washed. The resulting stained specimens were mounted in VectaShield Antifade Mounting Medium (CH-1000; Vector Laboratories, USA). Immunofluorescent images were captured using a microscope (Olympus Microscope System BX51; Olympus, Tokyo, Japan)^30^.

For H&E analysis, each mouse's head was removed and fixed in 4% paraformaldehyde. Serial 4 μM thick sections were cut on a paraffin microtome designed for histological analysis using H&E. For immunohistochemistry, paraffin sections were dewaxed, and incubated with 3% hydrogen peroxide at room temperature for 10 min to inactivate endogenous peroxidase. The sections were placed into an antigen retrieval solution (0.01 M citrate), repaired using a pressure cooker at 100 °C for 5 min and cooled to room temperature before washing five times with PBS for 5 min each. Sections were subsequently blocked with 1% bovine serum albumin at room temperature for 60 min and incubated overnight at 4 °C with anti‑mouse TLR4 monoclonal antibody at a dilution of 1:100. After washing, the sections were incubated with horse-radish peroxidase (HRP) at 37 °C in a humidified chamber for 60 min and subsequently washed five times with PBS for 5 min each. The sections were developed with 3′-Diaminobenzidine (DAB) and washed immediately with running water when brown particles appeared in the cytoplasm to terminate the reaction. The sections were secondarily stained with hematoxylin for 1 min^31^. For periodic acid‑schiff staining (PAS), at room temperature, paraffin-embedded tissue sections were serially dehydrated in xylene and ethanol. Then, sections were stained with PAS. Slides were incubated with 0.5% periodic acid solution for 5 min, then stained with Schiff's reagent for 15 min, followed by counterstaining with hematoxylin solution for 1 min^32^. Quantitative TLR4 and Myd88 colocalization analysis was done with JACoP tool (ImageJ software) using Pearson's correlation coefficient.

### Immunoprecipitation

Protein samples from airway epithelial cell extracts and mouse mucosa tissue cell extracts (500 μg protein) were immunoprecipitated by incubation with anti-TLR4 or MyD88 antibody (25 μg) overnight at 4 °C, followed by incubation with protein G-agarose bead (40 μL of a 1:1 slurry) for 1 h at room temperature. Immune complexes were washed five times with a RIPA buffer, boiled for 5 min in a Laemmli sample buffer, and analyzed by SDS-PAGE and immunoblotting with the indicated antibodies. The immunoprecipitation antibodies used were TLR4 (SC-293072, Santa Cruz Biotechnology, Santa Cruz, CA, USA) and MyD88.

### Proteome profiler antibody array analysis

The protein from airway epithelial cells and pHNECs was extracted using a RIPA lysis buffer. Proteome Profiler Human XL Cytokine Array Kit (#ARY022B) is membrane-based sandwich immunoassay. Following manufacturer recommendations, each membrane was incubated with 100 μL of pooled plasma overnight at 4 °C. Captured plasma proteins were detected with biotinylated detection antibodies and afterward, visualized with Streptavidin-HRP and chemiluminescent detection reagents. A signal was produced at each capture spot, proportionally with the amount of the protein-bound. The signal produced is proportional to the amount of analyte bound. The bands' intensities were analyzed using Quick Spots Image Analysis Software (Western Vision Software, http://www.wvision.com/QuickSpots.html)^33^.

### Real-time quantitative polymerase chain reactions

According to the manufacturer's instructions, total RNAs from the cells and mice nasal mucosa were extracted using TRIzol reagent (Thermo Fisher Scientific). AccuPower RT PreMix (Bioneer, Daejeon, Korea) was used for complementary DNA (cDNA) synthesis according to the manufacturer's instructions. The obtained cDNA was amplified with specific primers (Table 1). Polymerase chain reaction (PCR) was performed for cDNA synthesis using a T100 Thermal Cycler (Bio-Rad Laboratories, USA). The mRNA expression was analyzed using a CFX Connect Real-Time PCR Detection System (Bio-Rad Laboratories, USA) with PowerUp SYBR Green Master Mix (Applied Biosystems, USA). All PCR assays were performed in triplicate. The relative gene expression was analyzed using the 2^−∆∆Ct^ method^34^. The details of the primers are shown in Table 1.

**Table 1:** Details of the oligonucleotide sequences for qRT-PCR.

Oligonucleotides			
Gene	5′→3′	Human	Mouse
NLRP3	Forward	GGACTGAAGCACCTGTTGTGCA	TCACAACTCGCCCAAGGAGGAA
	Reverse	TCCTGAGTCTCCCAAGGCATTC	AAGAGACCACGGCAGAAGCTAG
Caspase-1	Forward	GCTGAGGTTGACATCACAGGCA	GGCACATTTCCAGGACTGACTG
	Reverse	TGCTGTCAGAGGTCTTGTGCTC	GCAAGACGTGTACGAGTGGTTG
IL-1β	Forward	CCACAGACCTTCCAGGAGAATG	TGGACCTTCCAGGATGAGGACA
	Reverse	GTGCAGTTCAGTGATCGTACAGG	GTTCATCTCGGAGCCTGTAGTG
NF-κB-p65	Forward	TGAACCGAAACTCTGGCAGCTG	TCCTGTTCGAGTCTCCATGCAG
	Reverse	CATCAGCTTGCGAAAAGGAGCC	GGTCTCATAGGTCCTTTTGCGC
IL-4	Forward	CCGTAACAGACATCTTTGCTGCC	ATCATCGGCATTTTAACGAGGTC
	Reverse	GAGTGTCCTTCTCATGGTGGCT	ACCTTGGAAGCCCTACAGACGA
IL-17	Forward	CGGACTGTGATGGTCAACCTGA	CAGACTACCTCAACCGTTCCAC
	Reverse	GCACTTTGCCTCCCAGATCACA	TCCAGCTTTCCCTCCGCATTGA
TNF-α	Forward	CTCTTCTGCCTGCTGCACTTG	GGTGCCTATGTCTCAGCCTCTT
	Reverse	ATGGGCTACAGCTTGTCACTC	GCCATAGAACTGATGAGAGGGAG
IFN-γ	Forward	GAGTGTGGAGACCATCAAGGAAG	CAGCAACAGCAAGGCGAAAAAGG
	Reverse	TGCTTTGCGTTGGACATTCAAGTC	TTTCCGCTTCCTGAGGCTGGAT
IRF3	Forward	TCTGCCCTCAACCGCAAAGAAG	
	Reverse	TACTGCCTCCACCATTGGTGTC	
IRF7	Forward	CACACACACATGCTGGACTC	
	Reverse	CCTTGGTTGGGACTGGATCT	

### IL-1β enzyme-linked immunosorbent assay

The supernatants from cultured A549, RPMI2650, and pHNECs were collected. Protein levels of IL-1β in the supernatants were detected using ELISA kits (SLB50, R&D Systems, Minneapolis, MN, USA). Assay diluent was added to each well with the standard and samples, and the wells were covered using an adhesive strip and incubated at room temperature. Each well was aspirated and washed as the complete removal of the liquid at each step is essential for best performance. Conjugate was added to each well, and the above steps were repeated. Plates were washed with a wash buffer, and substrate solution was added to each well and incubated for 30 min at room temperature. Notably, the procedure requires the samples to be protected from light. Once 1 mol/L sulfuric acid was added, the substrate reaction is halted, and the extinction was measured at a wavelength of 450 nM using a multi-plate ELISA reader^35^. According to the manufacturer's instructions, in mice NLF, IL-1β levels were detected using the ELISA kits (SMLB00C, R&D Systems).

### Isolation of cytosolic and nuclear fractions

Nuclear protein extracts from A549, RPMI2650 cells and pHNECs were prepared using the NE-PER kit (Thermo Fisher Scientific, USA) according to the manufacturer's instructions. After that, nuclear proteins (the supernatant) were used immediately or stored at − 80 °C.

### Statistical analysis

Data are expressed as mean ± standard error (SEM). The data were analyzed via a one-way analysis of variance (ANOVA) and post-hoc multiple variation comparisons (Tukey's HSD test). All data were analyzed using GraphPad Prism v.5.10 software (GraphPad Software Inc., San Diego, CA, USA). Statistical comparisons between the different treatments were performed using one-way ANOVA with the Tukey multiple comparison post-test. Data normality was tested by the D'Agostino-Pearson omnibus normality test when “n” was sufficient to do so. *P*-values of < 0.05 were considered as statistically significant).

## Results

### PVP-I suppress NLRP3- and NF-κB-dependent inflammatory pathways in airway epithelial cells

LPS, ATP, and PVP-I concentrations were selected up to 1000 ng/mL, 5 mM, and 0.1% for the following experiments, respectively (Supplementary Fig. S1a). Protein expression of NLRP3 were elevated in the LPS and ATP (LPS/ATP) treated cells compared with cells treated with LPS or ATP alone (Supplementary Fig. S1b). Expressions of NLRP3 mRNA were also significantly elevated in LPS/ATP treated cells compared with cells treated with each alone (Supplementary Fig. S1c).

**Figure 1:**
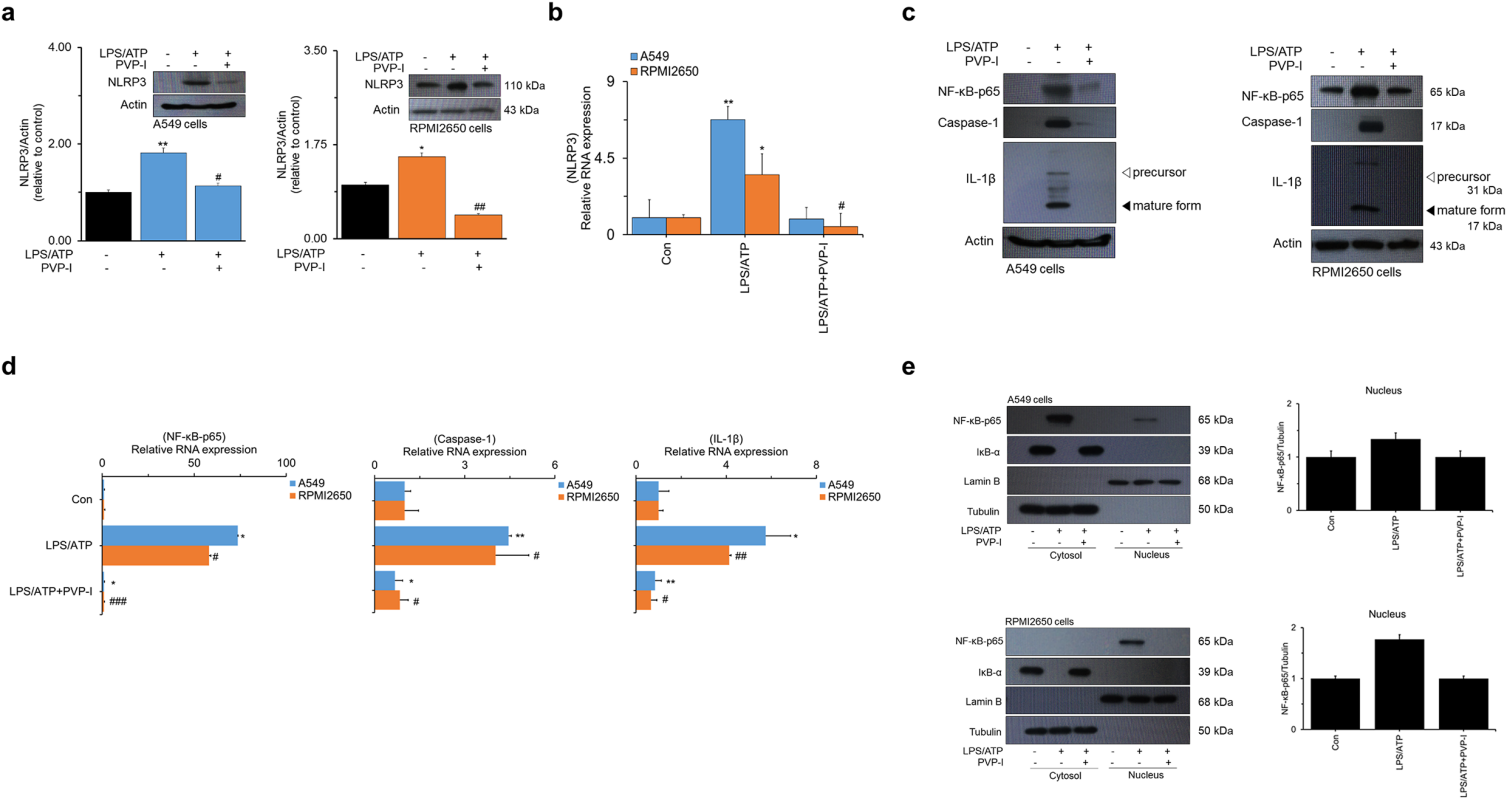
PVP-I suppressed LPS plus ATP induced activations of NLRP3 inflammasome response and associated signal molecules in airway epithelial cells (A549 and RPMI2650 cells). Treatment with PVP-I inhibited LPS/ATP-induced activation of NLRP3 inflammasome protein in A549 cells and RPMI2650 cells, as shown in (**a**) immunoblot and (**b**) qRT-PCR. Cells were stimulated with LPS and ATP for 24 h, and PVP-I (0.1%) was additionally added to the cells for 1 h (to produce an inflammation environment) after LPS and/or ATP stimulation. (**c**) and (**d**) Expression of NF-κB-p65, caspase-1 and IL-1β were also inhibited by PVP-I treatment in airway epithelial cells, as shown by immune blot and qRT-PCR. (**e**) Cytosolic and nuclear extracts were analyzed by immunoblot with indicated antibodies for NF-κB-p65 and IκB-α. Anti-Lamin B and anti-α-tubulin antibodies were used as loading controls for the nucleus and cytosol, respectively. Statistical significance: *P < 0.05 and **P < 0.01 compared with the control group; ^#^*P* < 0.05, ^##^*P* < 0 .01 and ^###^*P* < 0.01 compared with the LPS/ATP group.

**Figure 2:**
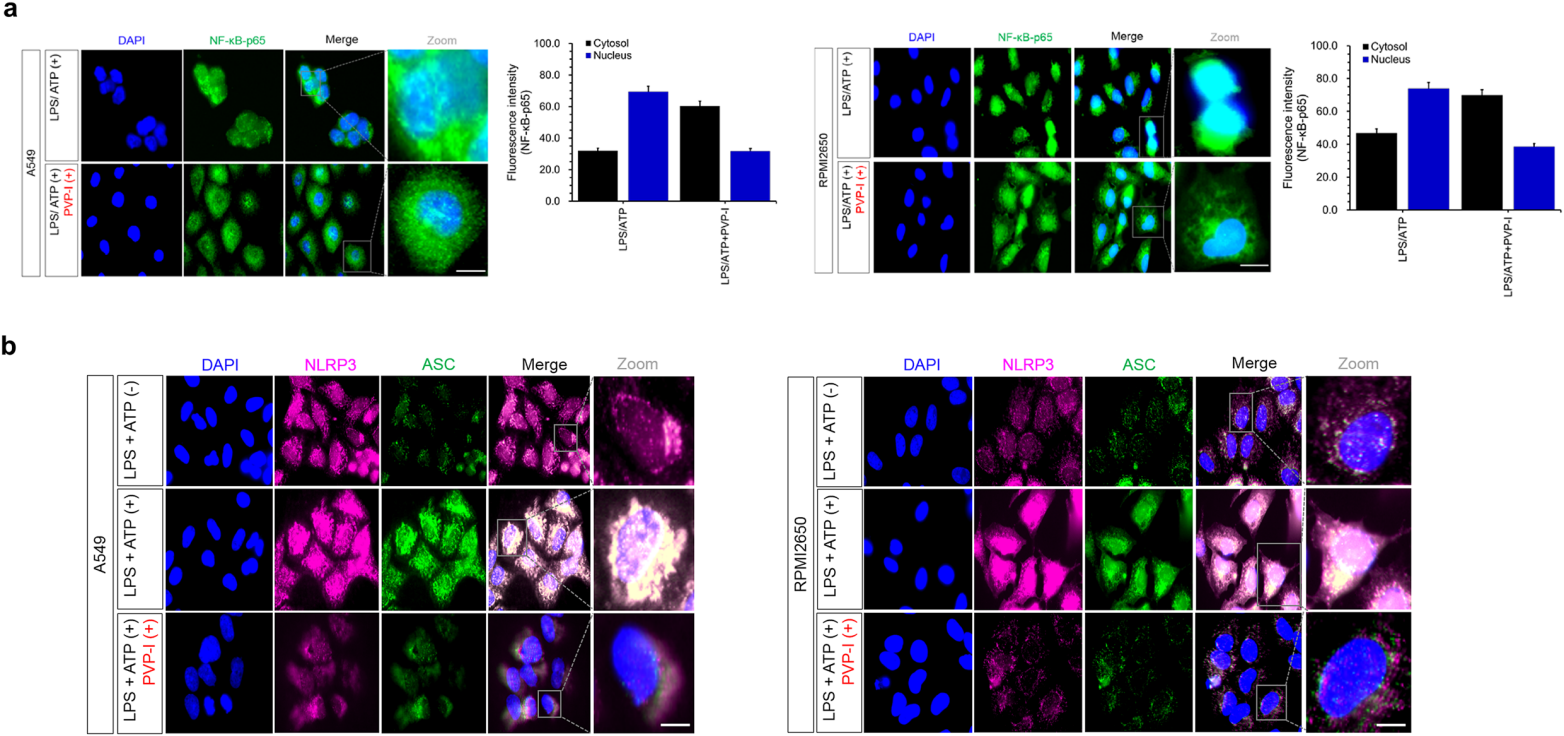
The effect of PVP-I on LPS plus ATP-induced translocation of NF-κB-p65, and activation of NLRP3 inflammasome response in airway epithelial cells. (**a**) Representative image of immunofluorescence on NF-κB-p65 (green) and DAPI (blue) in airway epithelial cells (A549 and RPMI2650 cells). LPS/ATP-induced NF-κB-p65 translocation to nucleus was inhibited by PVP-I treatment in airway epithelial cells. (**b**) Immunofluorescence for NLRP3 (red) and ASC (green) co-localization in airway epithelial cells. LPS/ATP-induced NLRP3/ASC complexes were inhibited by PVP-I treatment in these airway cells. The scale bar indicates 100 μm.

Protein and mRNA expression of NLRP3 induced by LPS/ATP was significantly decreased by PVP-I treatment in airway epithelial cells (Fig. 1a,b). NLRP3 inflammasome-associated molecules were also evaluated in these airway epithelial cells, and expression of NF-κB-p65, caspase-1, and IL-1β were strongly inhibited by PVP-I treatment in both protein and mRNA levels (Fig. 1c,d). Treatment of LPS/ATP allowed NF-κB-p65 translocation to the nucleus and IκB-α degradation in the cytosol, and these phenomena were reversed by treatment of PVP-I. Translocation of NF-κB-p65 from cytosol to nucleus was effectively decreased by PVP-I treatment in both A549 and RPMI2650 airway epithelial cells (Fig. 1e). Immunofluorescence analysis also showed that LPS/ATP-induced NF-κB-p65 translocation to the nucleus was inhibited by PVP-I treatment in airway epithelial cells (Fig. 2a). NLRP3/ASC assemblies in the cytoplasm were also induced by LPS/ATP treatment, and were inhibited by PVP-I treatment in airway epithelial cells (Fig. 2b).

**Figure 3:**
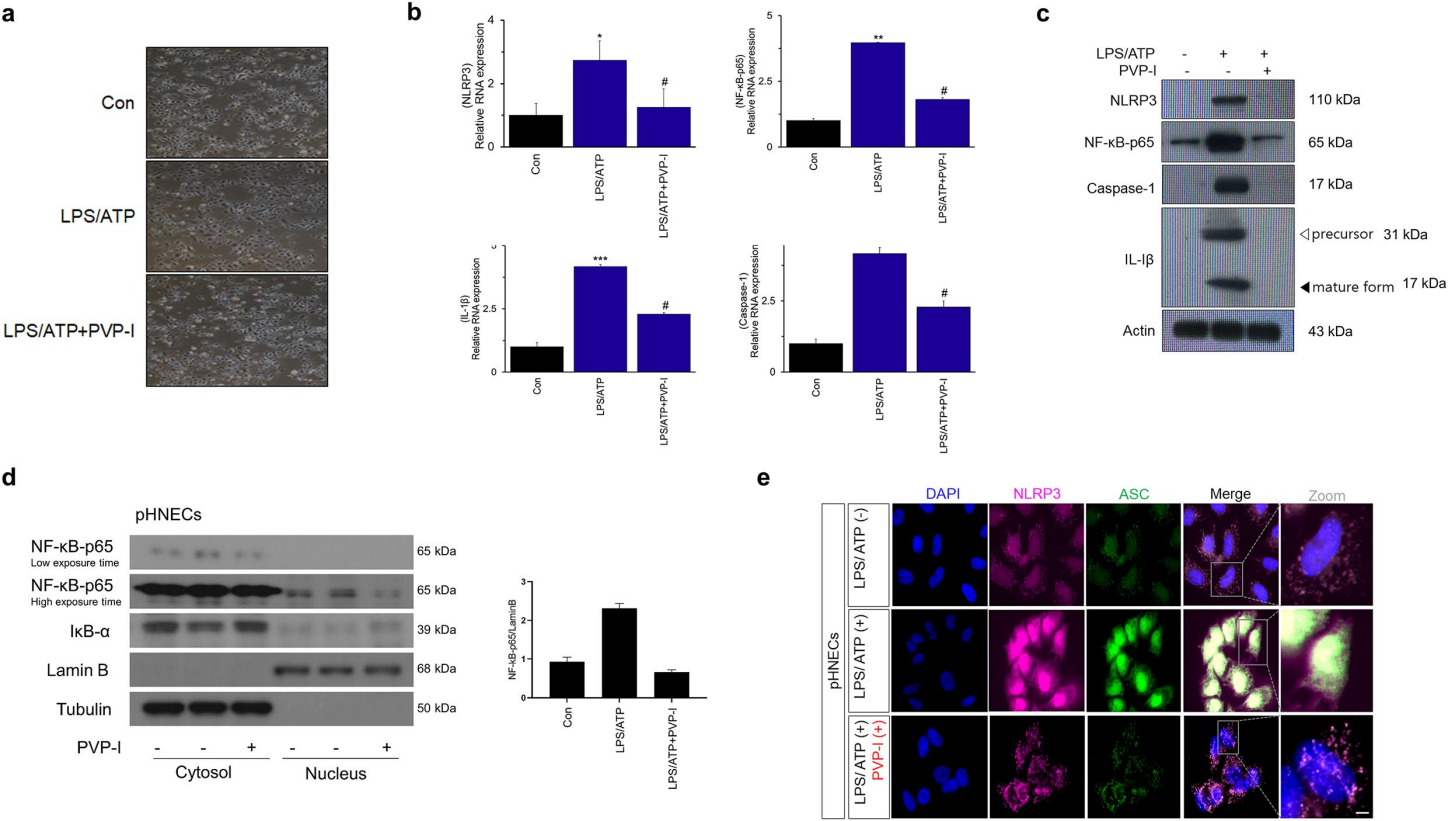
Alteration of inflammation molecules in primary cultured cells from nasal polyp patients. (**a**) Optical microscope of cell viability in the human nasal epithelial primary cells (pHNECs) from nasal polyp patients. Primary cultured cells from nasal polyp patients were treated with LPS (1000 ng/mL), ATP (5 mM) and PVP-I (0.1%) for 24 h. Representative photomicrographs of cell morphology was observed using an optical microscope. (**b**) and (**c**) mRNA and protein expression of NLRP3, NF-κB-p65, caspase-1 and IL-1β in pHNECs treated with LPS, ATP with or without PVP-I, as shown by qRT-PCR and immunoblot. Statistical significance: *P < 0.05, **P < 0.01 and ***P < 0.001 compared with the control group; ^#^*P* < 0.05 compared with the LPS/ATP group. (**d**) Western blot assay for NF-κB nuclear translocation and IκB-α levels in pHNECs. (**e**) Immunofluorescence for NLRP3 (red) and ASC (green) co-localization in pHNECs. The scale bar indicates 100 μm.

Primary cultured cells from nasal polyp patients were treated with LPS (1000 ng/mL), ATP (5 mM) and PVP-I (0.1%) for 24 h. Cell morphology was observed using an optical microscope. Morphologic changes were not observed at the indicated concentrations of reagents when compared to the control (media alone) in optical microscopic images of the pHNECs. (Fig. 3a). PVP-I treatment decreased LPS+ ATP-induced expressions (both protein and mRNA) of p65, NLRP3, caspase-1 and IL-1β in pHNECs (Fig. 3b,c). PVP-I treatment in pHNECs inhibited NF-κB-p65 translocation to the nucleus that LPS/ATP induced (Fig. 3d). This is also a similar result with respect to A549 and RPMI2650 cells. NLRP3/ASC assemblies in the cytoplasm were also induced by LPS/ATP treatment, and were inhibited by PVP-I treatment in pHNECs (Fig. 3e). LPS/ATP-induced expressions of NLRP3, NF-κB-p65, caspase-1, and IL-1β, and NLRP3/ASC assemblies were suppressed by PVP-I treatment in pHNECs (Fig. 3b–e). These results indicated that PVP-I treatment could inhibit the activation of NLRP3 inflammasome and associated molecules in airway epithelial cells and pHNECs.

### PVP-I inhibits LPS/ATP-induced cytokine production in airway epithelial cells

**Figure 4:**
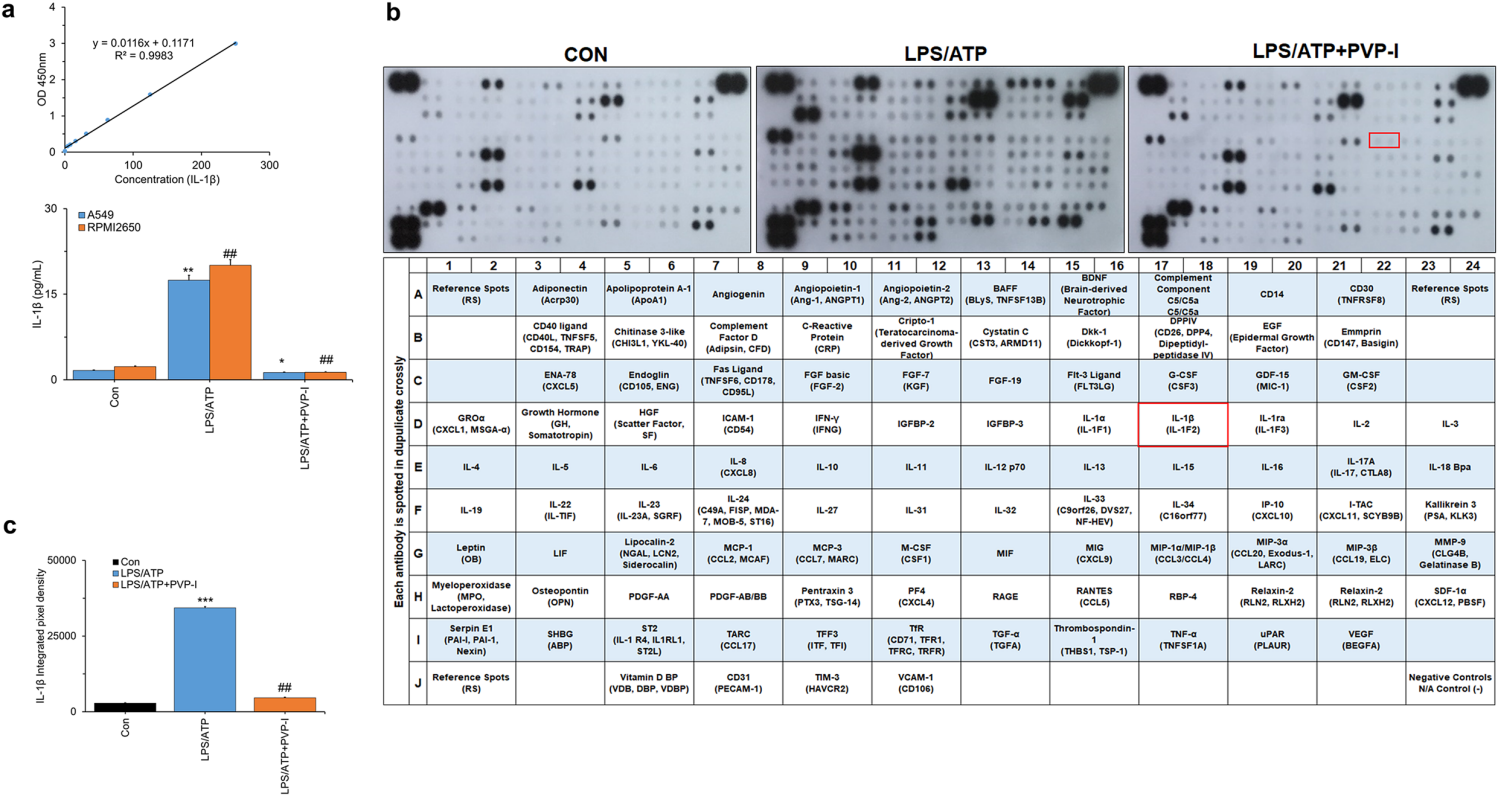
Profiling proteins by inflammation response in airway epithelial cells. Inhibition of LPS and ATP-induced IL-1β secretion by PVP-I. Airway epithelial cells were treated with PVP-I (0.1%) for 1 h after LPS (1000 ng/mL) and ATP (5 mM) treatment. (**a**) After 24 h-incubation, the airway epithelial cell culture medium was assayed using an ELISA for IL-1β. Statistical significance: *P < 0.05 and **P < 0.01 compared with the control group; ^##^*P* < 0 .01 compared with the LPS/ATP group (**b**) After 24 h-induction, the pHNECs were subjected to cytokine antibody array assay. Each proteins represented by duplicate spots in the respective membrane. IL-1β protein is boxed in red. (**c**) Data were analyzed using the Quick Spots Image Analysis Software. Statistical significance: ***P < 0.001 compared with the control group; ^##^*P* < 0.01 compared with the LPS/ATP group.

LPS/ATP-induced production of IL-1β was markedly reduced by PVP-I treatment in airway epithelial cells (A549 and RPMI2650, Fig. 4a). In addition, PVP-I treatment reduced the NF-κB signaling related cytokines (APoA1, Ang-1, BAFF, CD40L, EGF, Fas L, GM-CSF, GROα, CD54L, IFN-γ, IL-1α, IL-1ra, IL-4, IL-6, IL-8, IL-10, IL-12p70, IL-17A, IL-23, IP-10, MCP1, M-CSF, MMP9, TFF3, TNF-α, VESF, and VCAM-1) induced by LPS/ATP stimulation in pHNECs (Fig. 4b and Supplementary Fig. S3). LPS/ATP-induced IL-1β cytokine production was also significantly decreased by PVP-I treatment in pHNECs (red box in Fig. 4b,c). We also checked the MyD88-independent pathway which is related with activation of IFN regulatory factor (IRF)3 and IRF7 activation. LPS/ATP-induced activation of these transcription factors were significantly decreased by PVP-I treatment in A549 and pHNECs (Supplementary Fig. S4a,b). These results indicated that PVP-I could strongly inhibit both MyD88-dependent and -independent pathway and thus suppress the production of inflammatory or pro-inflammatory cytokines.

### PVP-I suppresses LPS/ATP-induced inflammatory response by blocking TLR4 signaling

**Figure 5:**
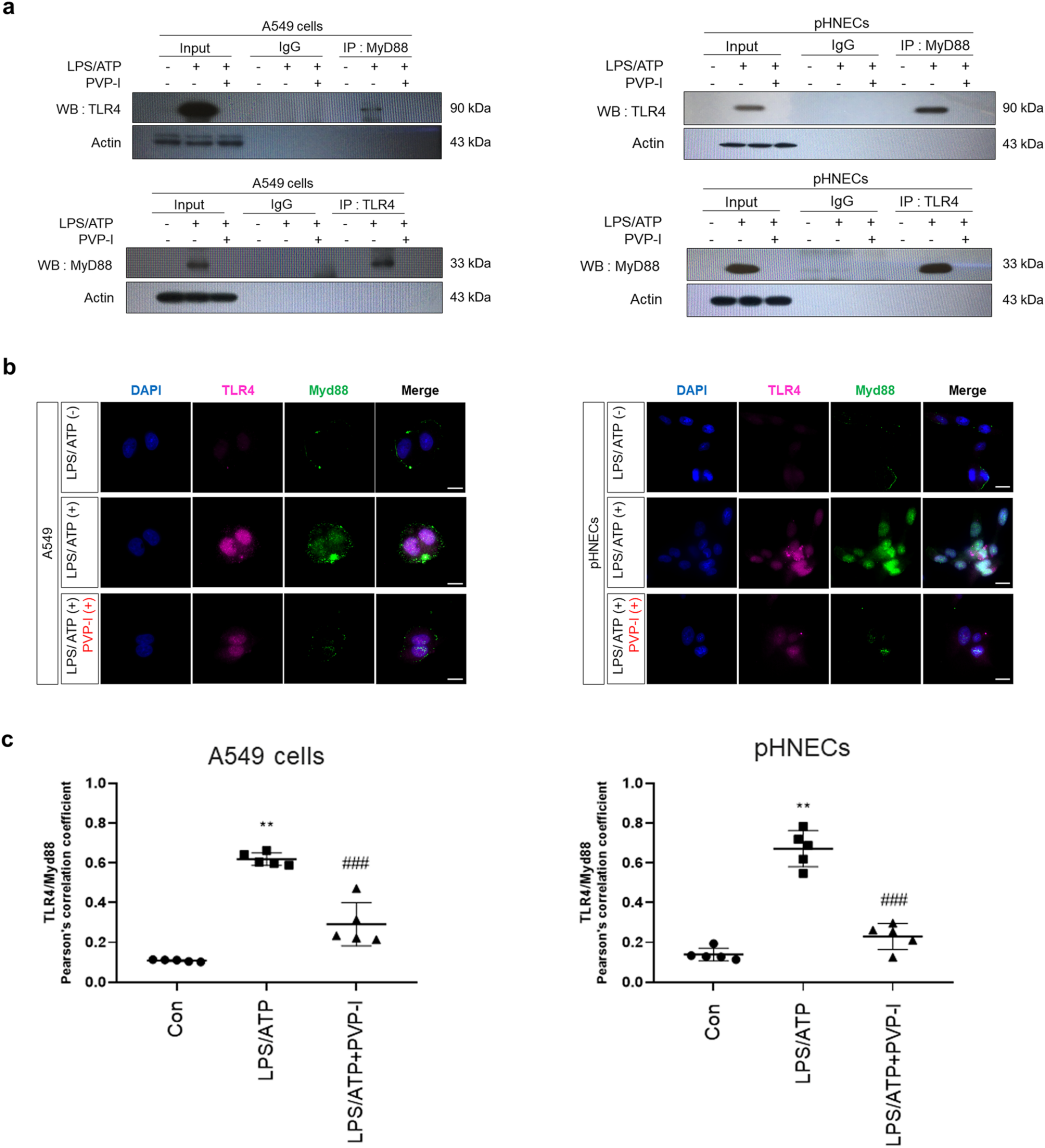
PVP-I inhibits TLR4 and MyD88 co-localization which were induced by inflammatory response. Airway epithelial cells were pre-treated with LPS (1000 ng/mL) and ATP (5 mM) and then exposed to 0.1% PVP-I. (**a**) The cells lysate were immunoprecipitated with anti- MyD88 and TLR4 antibody, and then immunoprecipitated proteins were detected using anti-TLR4 and anti-MyD88 antibodies. The pHNECs had the same experimental methods as the airway epithelial cells. PVP-I was found to inhibit the co-localization with TLR4 and MyD88 complex formation. Actin was used as a control for input. Airway epithelial cells were pre-treated with LPS and ATP for 60 min and then treated or not with PVP-I for 24 h. (**b**) Immunocytochemistry for TLR4 (red) and MyD88 (green) co-localization in A549 cells and pHNECs is shown. Increased expressions of TLR4 and MyD88 by LPS/ATP stimulation were inhibited by PVP-I treatment in A549 cells and pHNECs. The scale bar indicates 10 μm. Airway epithelial cells were pre-treated with LPS and ATP for 10 min and then treated or not with PVP-I for 10 min. (**c**) TLR4 and Myd88 colocalization was assessed by calculating the Pearson correlation coefficient on twenty images per condition using the JACoP plugin on ImageJ.

The binding of TLR4 and MyD88 are crucial for signaling inflammatory response^41^. The formation of TLR4 and MyD88 was increased after LPS/ATP stimulation, and treatment of PVP-I inhibited the intensity of the TLR4 band immunoprecipitated with an anti-MyD88 protein. The reverse immunoprecipitation of MyD88 with TLR4 antibody also showed blocking of complex formation after PVP-I treatment (Fig. 5a). Double immunofluorescent study also showed that LPS/ATP-induced expressions of TLR4 and MyD88 were suppressed in A549 cells and pHNECs (Fig. 5b). We conducted the Pearson’s R correlation analysis to prove the co-localization between TLR4 and MyD88 expression in A549 cells and pHNECs using Pearson’s correlation coefficient, and there were positive correlations between TLR4 and MyD88 expression determined by Pearson analysis (Fig. 5c). These results indicated that PVP-I could inhibit TLR4/MyD88 complex formation, which is induced by LPS/ATP stimulation. We wondered whether PVP-I could also affect the expression of other TLRs on airway epithelial cells. The results of RT-PCR indicated that elevated expressions of TLRs on airway epithelial cells were inhibited by PVP-I treatment (Supplementary Fig. S5). Therefore, PVP-I seems to non-specifically inhibit surface receptors of airway epithelial cells which were induced by stimulation of various pathogens.

### PVP-I inhibits mucosal inflammation in mice with CRS

**Figure 6:**
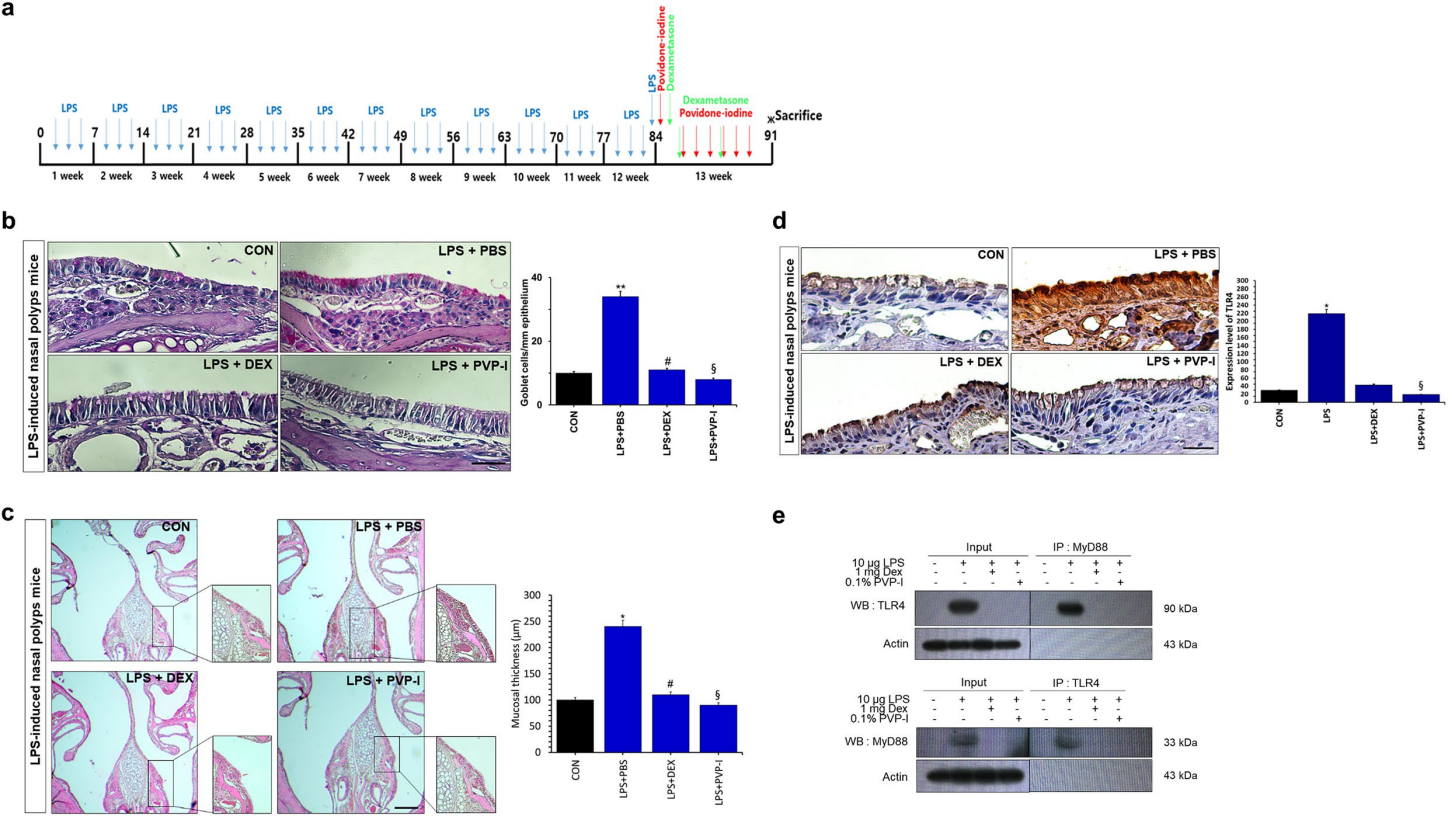
PVP-I inhibits inflammation in LPS-stimulated mice. (**a**) Nasal polyps mouse models protocol. Mice were assigned to 4 groups and administered saline or PVP-I at doses of 0.1% intra-nasally for on 7 days after LPS administration (intra-nasal) for 12 weeks. Nasal polyps mouse models protocol. Mice were assigned to 4 groups and administered PVP-I at doses of 0.1% intra-nasally or DEX at doses of 1 mg/kg intraperitoneally for on 7 days after 10 µg LPS intra-nasally for 12 weeks. (**b**) Representative hematoxylin and eosin staining to demonstrate nasal mucosa tissue. Statistical significance: *P < 0.05 compared with the control group; ^#^*P* < .05 compared with the LPS + PBS group; ^§^*P* < 0.05 compared with the LPS + DEX group. (**c**) Goblet cells increased in the epithelium with PAS staining upon LPS administration. In the epithelium, number of goblet cells of the LPS administration group was more than in the CON group and was further inhibited in the PVP-I treatment group. Statistical significance: **P < 0.01 compared with the control group; ^#^*P* < 0.05 compared with the LPS + PBS group; ^§^*P* < 0.05 compared with the LPS + DEX group. (**d**) Immunohistochemistry showing the TLR4 and MyD88 in the nasal mucosa of mice in each group. Statistical significance: *P < 0.05 compared with the control group; ^§^*P* < 0.05 compared with the LPS + DEX group. (**e**) Cells lysate in murine nasal mucosa tissue were immunoprecipitation with anti- MyD88 and TLR4 antibody, and then immunoprecipitated proteins were detected using anti-TLR4 and anti-MyD88 antibodies. PVP-I was found to inhibit the TLR4 and MyD88 complex formation. Actin was used as a control for input.

**Figure 7:**
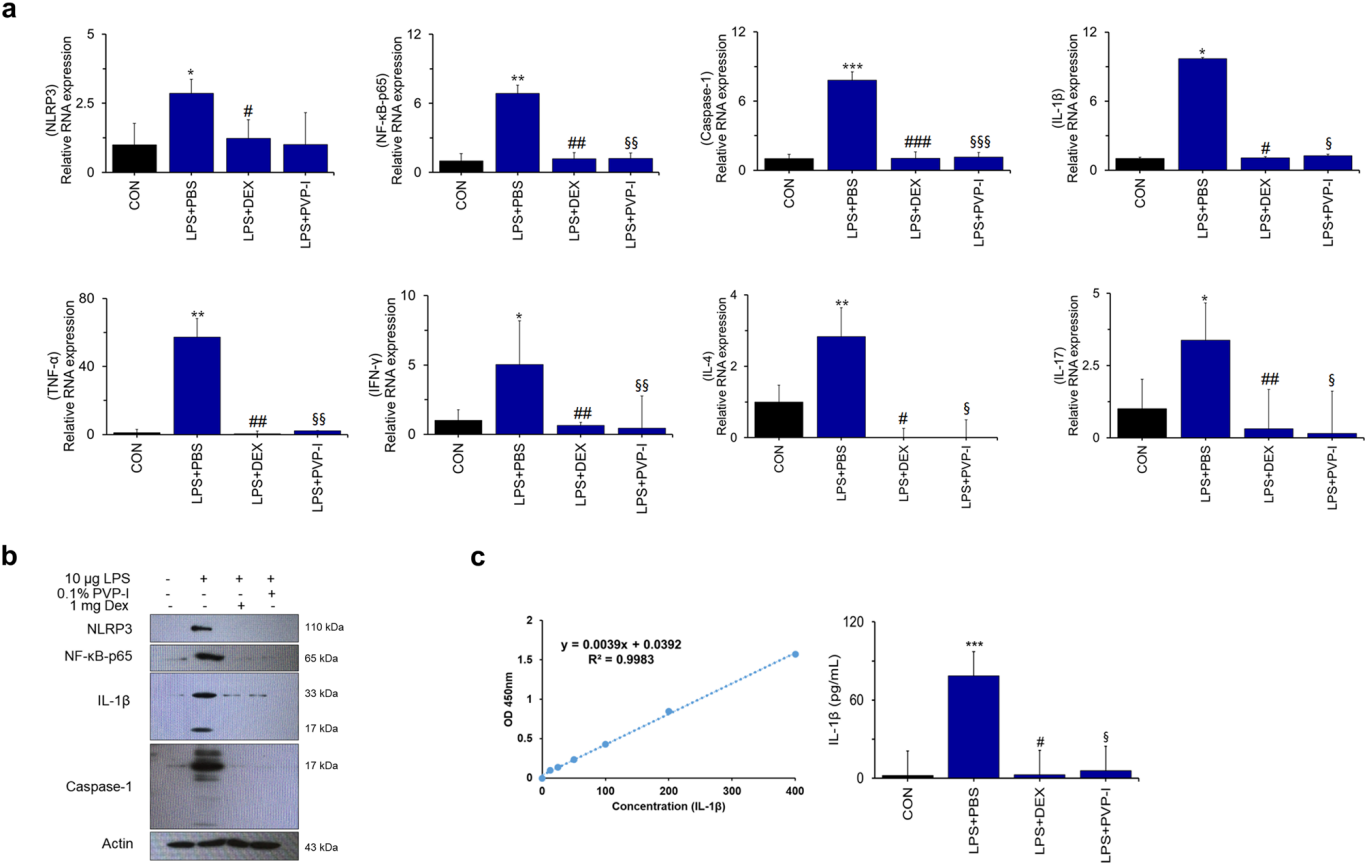
PVP-I inhibits TLR4 and MyD88 complex formation in nasal polyps mouse model. (**a**) Bar graphs represent the relative expression of cytokines. Statistical significance: *P < 0.05, **P < 0.01 and ***P < 0.001 compared with the control group; ^#^*P* < 0.05, ^##^*P* < 0.01 and ^###^*P* < 0.001 compared with the LPS + PBS group; ^§^*P* < 0.05, ^§§^*P* < 0.01 and ^§§§^*P* < 0.001 compared with the LPS + DEX group. (**b**) The expression of NLRP3, NF-κB-p65, caspase-1 and IL-1β were measured using an immunoblot in murine nasal mucosa tissues. Actin protein was used as a loading control. (**c**) Production of IL-1β in the nasal lavage fluid (NLF) were measured using the ELISA assay. Statistical significance: ***P < 0.001 compared with the control group; ^#^*P* < 0.05 compared with the LPS + PBS group; ^§^*P* < 0.05 compared with the LPS + DEX group.

Former studies revealed that NLRP3 and IL-1β are highly expressed in NPs from subjects with CRSwNP, and are significantly correlated with neutrophilic nasal polyps^16–19^. Therefore, we investigated whether PVP-I can suppress the mucosal inflammation of neutrophilic CRS, which is related with increased expressions of NLRP3 inflammasome and associated molecules and inflammatory cytokines. Mucosal thickness (H&E stain, Fig. 6b) and the number of goblet cells (PAS stain, Fig. 6c) were significantly decreased in the groups of dexamethasone (LPS + DEX) or PVP-I treatment (LPS + PVP-I) compared with the PBS treatment group (LPS + PBS). TLR4 expression among groups was significantly higher in the LPS + PBS group compared with other groups (Fig. 6d). TLR4/MyD88 complex formation stimulated with LPS was inhibited by PVP-I or dexamethasone treatment in the nasal mucosa (Fig. 6e). NF-κB-p65, caspase-1, and IL-1β were significantly inhibited by PVP-I treatment (LPS + PVP-I) or dexamethasone (LPS + DEX) (Fig. 7a,b). The mRNA expressions of TNF-α, IFN-γ, IL-4, and IL-17 were significantly higher in LPS-induced CRS mice (LPS + PBS) compared with those of control group mice, and these cytokines were significantly decreased by PVP-I (LPS + PVP-I) and dexamethasone (LPS + DEX) treatment (Fig. 7a). In addition, IL-1β production in NLF was significantly decreased in the mice treated with dexamethasone or PVP-I (Fig. 7c). These results indicated that topical instillation of PVP-I in mice alleviates mucosal inflammation of LPS-induced CRS mice.

## Discussion

Inflammatory responses exert their effect on cells mainly through the NF-κB signaling^18^. We found that PVP-I inhibited several molecules' expression in this signaling, such as NLRP3, NF-κB-p65, caspase-1, and IL-1β in LPS/ATP-induced airway epithelial cells and pHNECs. (Supplementary Figs. S1 and S2). Previous studies have reported anti-inflammatory mimic effects of PVP-I^23–26^. However, the underlying molecular mechanisms of PVP-I on anti-inflammatory effects were not revealed. To our knowledge, this is the first study that evaluates the underlying mechanisms of PVP-I on inflammation in airway epithelial cells.

We tried verifying the anti-inflammatory effect of PVP-I using the NLRP3 inflammasome signal model in vitro. Recent studies have shown that NLRP3 inflammasome plays a pivotal role in various diseases, such as CRS, asthma, obstructive pulmonary disease, inflammatory bowel disease, metabolic disorders, multiple sclerosis, and other auto-immune and auto-inflammatory diseases^36–46^. Our results demonstrated that PVP-I effectively suppressed NLRP3 inflammasome-associated molecules and cytokines, activated by LPS/ATP stimulation.

To inhibit the inflammatory signal inside airway epithelial cells, free iodine may be able to enter the cytosol through the cell membrane. This allowed the destruction of cellular components, including enzymes essential to survive. Because 0.1% of PVP-I did not show cellular toxicity, other mechanisms may be involved in suppressing cellular signaling molecules and inflammatory cytokines in this study. Our findings suggest that PVP-I could inhibit the inflammatory response via TLR4 and MyD88 complex formation. Free iodine released by PVP-I is a strong oxidizing agent, and there is a possibility that free iodine destroys some parts of TLR4 and disturbs joining with MyD88; thus, blocking signal transmission. Indeed, TLR4 induced by LPS was suppressed by PVP-I treatment in airway epithelial cells (Fig. 5a,b).

A study indicated that NLRP3 inflammasome is more associated with non-eosinophilic CRS. Because the PVP-I target was thought to be TLR4 in vitro, expressions of TLR4 and TLR4-related signal molecules were evaluated in mice^47–50^, and these were decreased in PVP-I treated mice. Interestingly, IL-4 expression in mice nasal mucosa was also suppressed after PVP-I treatment. Future studies investigating PVP-I-induced suppression of PRR and other surface receptors are required.

Increased expressions of several types of cytokines were known to be involved in nasal polyp formation in patients with CRSwNP^7^. IRF3 and IRF7, which are associated with MyD88-independent pathway of TLR4 signalling, also play crucial roles in development of autoimmune disease or CRSwNP, and these molecules are thought to act as pro-inflammatory transcription factors in these diseases^51–53^. Our results showed that PVP-I can inhibit the inflammation-induced IL-1β secretion and IRF3/7 activation, and 27 cytokines in airway epithelial cells and pHNECs from nasal polyps (Fig. 4 and Supplementary Fig. S3).

In the pathogenesis of CRSwNP in Asian patients, NF-κB-associated inflammatory cytokines are a key factor and thought of as a therapeutic target^54^. This finding suggests that the PVP-I strongly inhibited inflammation signaling through NF-κB related molecule, such as IL-1β, and PVP-I may be used as a possible therapeutic remedy in CRS, especially non-eosinophilic CRS.

The therapeutic effects of PVP-I were similar to those of dexamethasone in our study. Anti-inflammatory effects of dexamethasone have been well described^55^. Dexamethasone showed its effect by inhibiting NF-κB and other inflammatory transcription factors and promoting anti-inflammatory genes^56^. Although the effects of dexamethasone were similar to those of PVP-I in this study, these drugs' target and route were quite different. Generally, the side effect of PVP-I is less serious than those of dexamethasone even when used long term.

Here, PVP-I was the first used to study an anti-inflammatory response by inhibiting the goblet cells in the NPs mouse models' mucosa tissue. In this study, PVP-I ameliorated goblet cell hyperplasia in neutrophilic CRS mice. Although we did not conduct an animal study in the eosinophilic mice model, PVP-I may effectively suppress mucosal inflammation, including goblet cell hyperplasia because PVP-I blocked the inflammation-related cytokines, such as TNF-α, IL-1β, and IL-4 in mucosa tissue of NPs mice models (Fig. 7a,b). Future studies are required to verify these effects in the eosinophilic CRS model. A study also demonstrated that diluted topical PVP-I rinses showed notable symptom improvement, especially discharge score in recalcitrant CRS, without affecting thyroid function, mucociliary clearance, or olfaction^57^.

Treatment of LPS/ATP allowed NF-κB-p65 translocation to the nucleus and IκB-α degradation in the cytosol. In RPMI2650 cells (Fig. 1e), we presume that all cytosolic NF-κB-p65 moved into the nucleus because the time spent between the treatment of LPS/ATP and the time the cells were harvested. In addition, we think the results can also be different according to the cell condition. That’s why we conducted this experiment with 3 different kinds of airway epithelial cells. RPMI2650 cell showed somewhat inconsistent results compared to other cells (A549 cells or pHNECs) in each experiment.

In summary, we demonstrated that PVP-I significantly attenuated inflammatory molecules and cytokines via blocking the formation of TLR4 and MyD88 complexes in the LPS-induced mucosal inflammation in non-eosinophilic CRS. We believe PVP-I could modulate critical inflammatory signals, and the need to study PVP-I-related signal inhibition on the cell surface receptors or molecules, such as pattern-recognition receptors (Supplementary Fig. S5). However, future studies will be needed to examine the protein expression of each receptor. We also believe that ACE2 (angiotensin-converting enzyme 2) and TMPRSS2 (transmembrane protease serine subtype 2) could be a target for PVP-I rinses of the upper airway.

## Supplementary Information

Supplementary Legends.

Supplementary Figure 1.

Supplementary Figure 2.

Supplementary Figure 3.

Supplementary Figure 4.

Supplementary Figure 5.

Supplementary Information.
