# Supplementary material for: Povidone iodine suppresses LPS-induced inflammation by inhibiting TLR4/MyD88 formation in airway epithelial cells
Source: Sci Rep. 2022 Mar 7;12:3681. doi: 10.1038/s41598-022-07803-2 (PMC8901750; doi:10.1038/s41598-022-07803-2)
Supplement: Supplementary file 1 — Supplementary Legends. [file 41598_2022_7803_MOESM1_ESM.docx]

**Supplementary Figure Legends**

**Supplementary Figure 1. Cell viability of LPS, ATP and PVP-I in airway epithelial cells.**

Effect of LPS, ATP and PVP-I on airway epithelial cells (A549, RPMI2650 cells). **a** After serum starvation, cells were treated with various concentration of LPS, ATP and PVP-I for 24 h. The viability of the cells were measured by the MTT assay. **b** and **c** LPS/ATP induced changes of NLRP3 protein and mRNA levels in airway epithelial cells. Cells were treated with LPS 1000 ng/mL and ATP 5 mM for 24 h. Statistical significance: **P* <.05 compared with the control group; ##*P* <.01 compared with the LPS group; §§<.01, §§§<.001 compared with the ATP group.

**Supplementary Figure 2. Localization of NLRP3 in airway epithelial cells.** Airway epithelial cells were treated with LPS (1000 ng/mL) and ATP (5 mM) for 60 min. **a** After 24 h-indubation, immunofluorescence for NLRP3 (red) localization in A549 cells. **b** After 24 h-indubation, immunofluorescence for NLRP3 (red) localization in RPMI2650 cells. The scale bar indicates 100 μm.

**Supplementary Figure 3. Inflammatory cytokines with NF-κB in pHNECs.** pHNECs were treated with PVP-I (0.1%) for 1 h after LPS (1000 ng/mL) and ATP (5 mM) treatment. After 24 h-incubation, the pHNECs culture medium was assayed using an ELISA for inflammaroty cytokines-related NF-κB signaling. Densitometric ratios of the array showed differences in the inflammation cytokines.

**Supplementary Figure 4. IFN regulatory factor (IRF)-3 and IRF-7 upon PVP-I in airway epithelial cells and pHNECs.** **a** Airway epithelial cells and **b** pHNECs were treated with PVP-

I (0.1%) for 1 h after LPS (1000 ng/mL) treatment. After 24 h-incubation, the RNA expression was assayed using a qRT-PCR.

**Supplementary Figure 5. Toll like receptors expression upon PVP-I in airway epithelial cells and pHNECs**. Airway epithelial cells and pHNECs were treated with PVP-I (0.1%) for 1 h after LPS (1000 ng/mL), SEB (500 ng/mL; TLR1, 2 and 6 stimulator), Poly(I:C) (5 μg/mL; TLR3 stimulator) and HMGB1 (500 ng/mL; TLR5 stimulator) treatment. After 24 h-incubation, the RNA expression was assayed using a qRT-PCR.

**Supplementary Figure 1**
