# Supplementary figures and images for: Povidone iodine suppresses LPS-induced inflammation by inhibiting TLR4/MyD88 formation in airway epithelial cells
Source: Sci Rep. 2022 Mar 7;12:3681. doi: 10.1038/s41598-022-07803-2 (PMC8901750; doi:10.1038/s41598-022-07803-2)

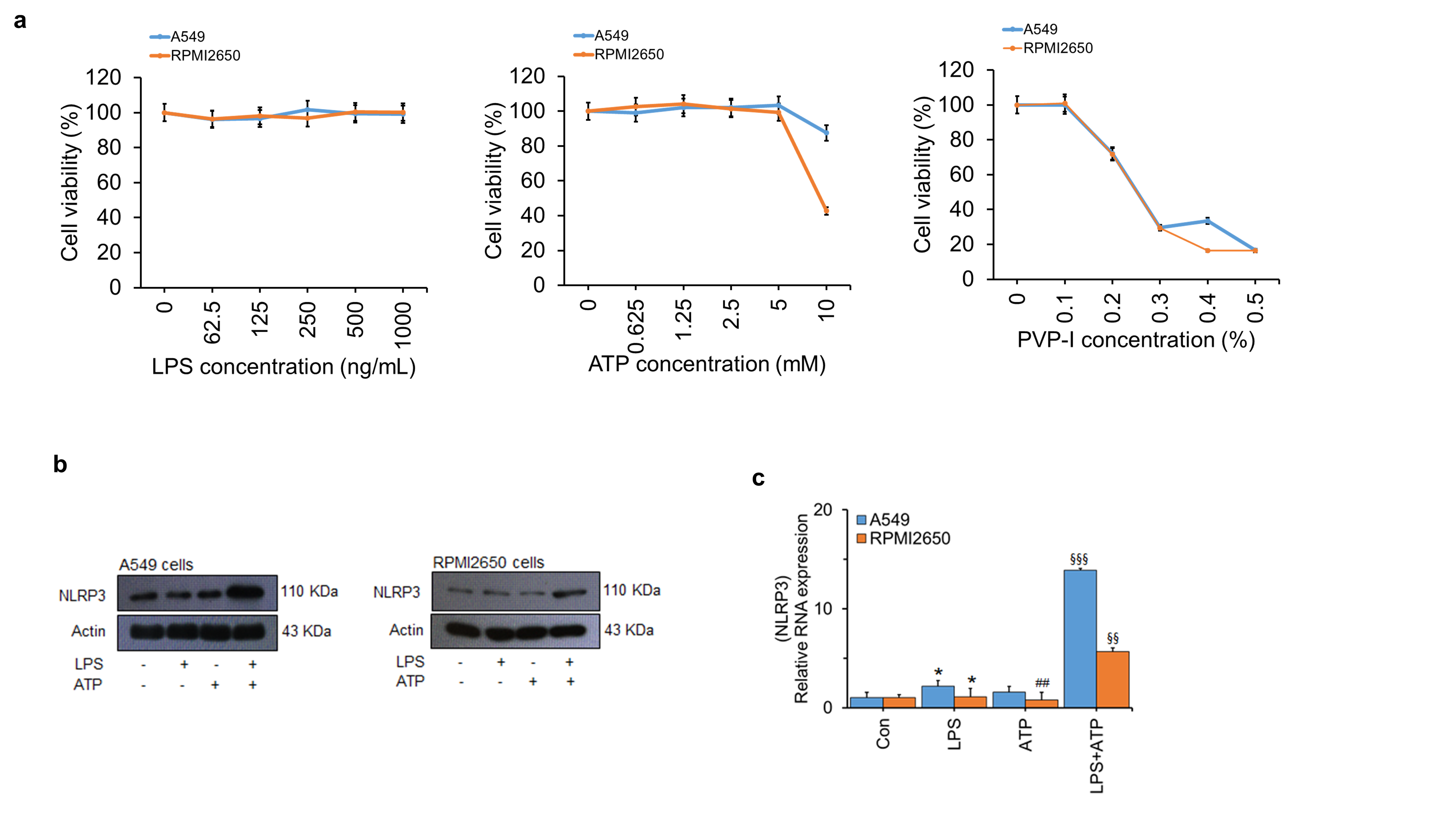

Supplement: Supplementary file 2 — Supplementary Figure 1. [file 41598_2022_7803_MOESM2_ESM.tif]

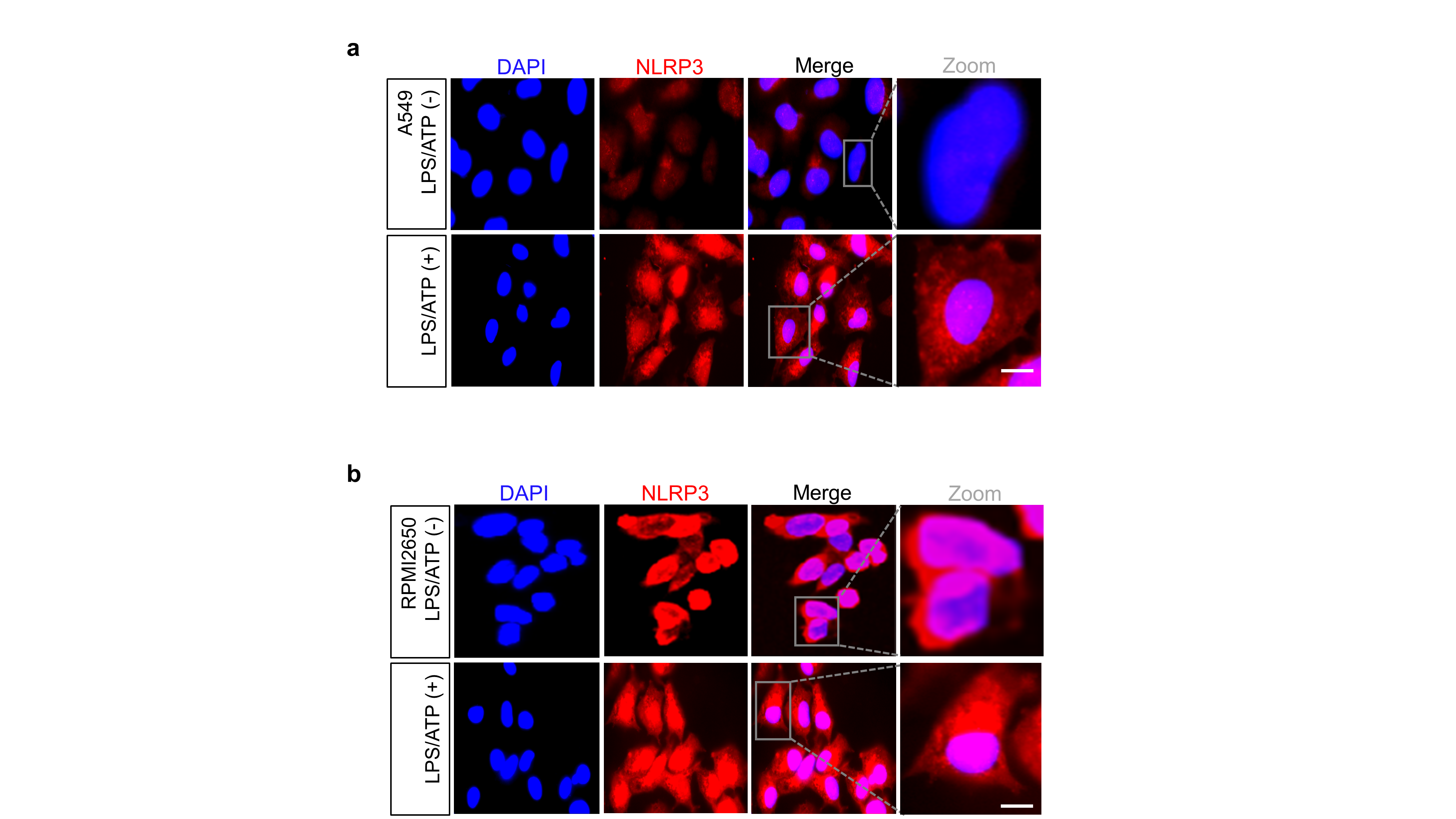

Supplement: Supplementary file 3 — Supplementary Figure 2. [file 41598_2022_7803_MOESM3_ESM.tif]

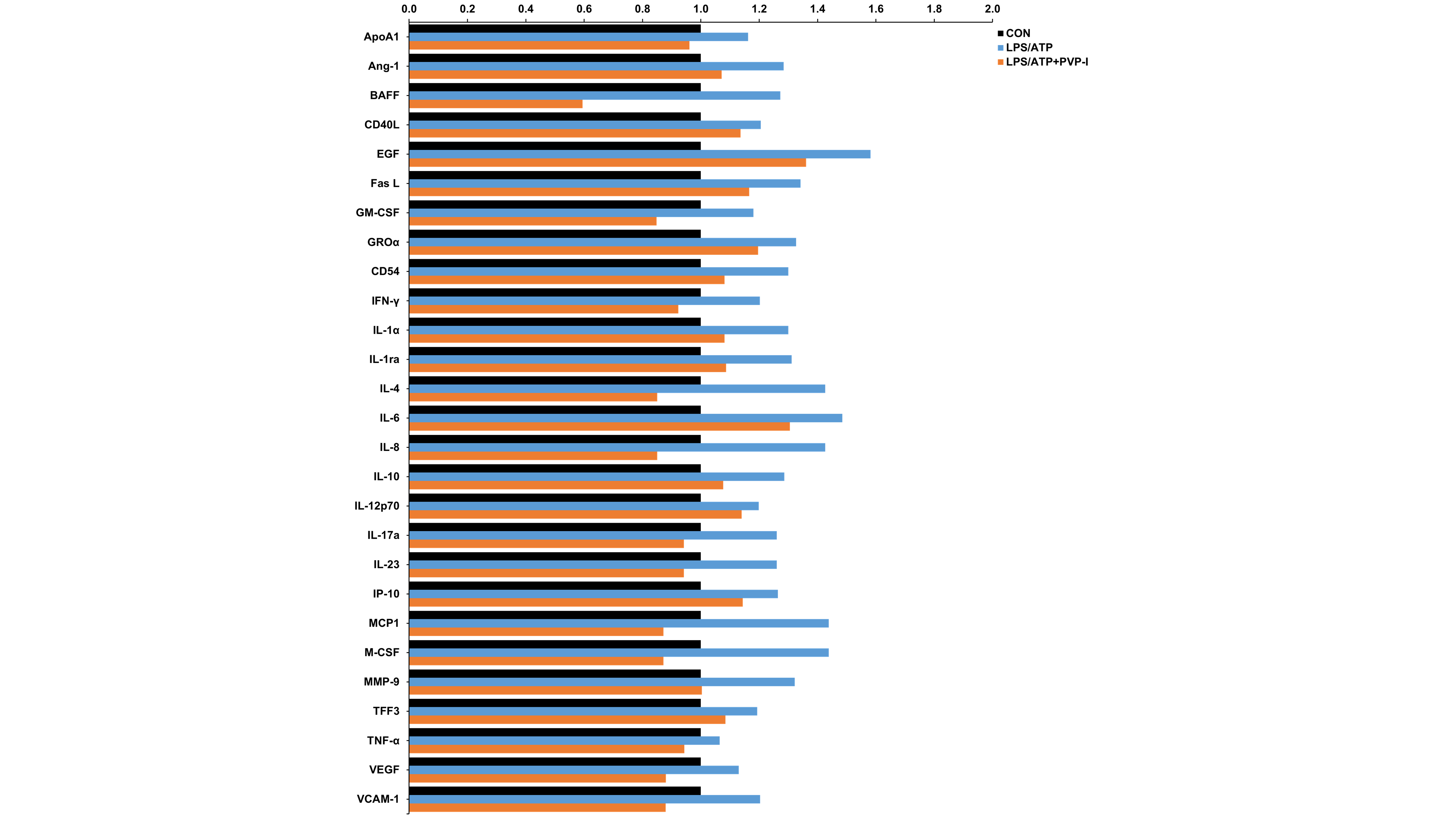

Supplement: Supplementary file 4 — Supplementary Figure 3. [file 41598_2022_7803_MOESM4_ESM.tif]

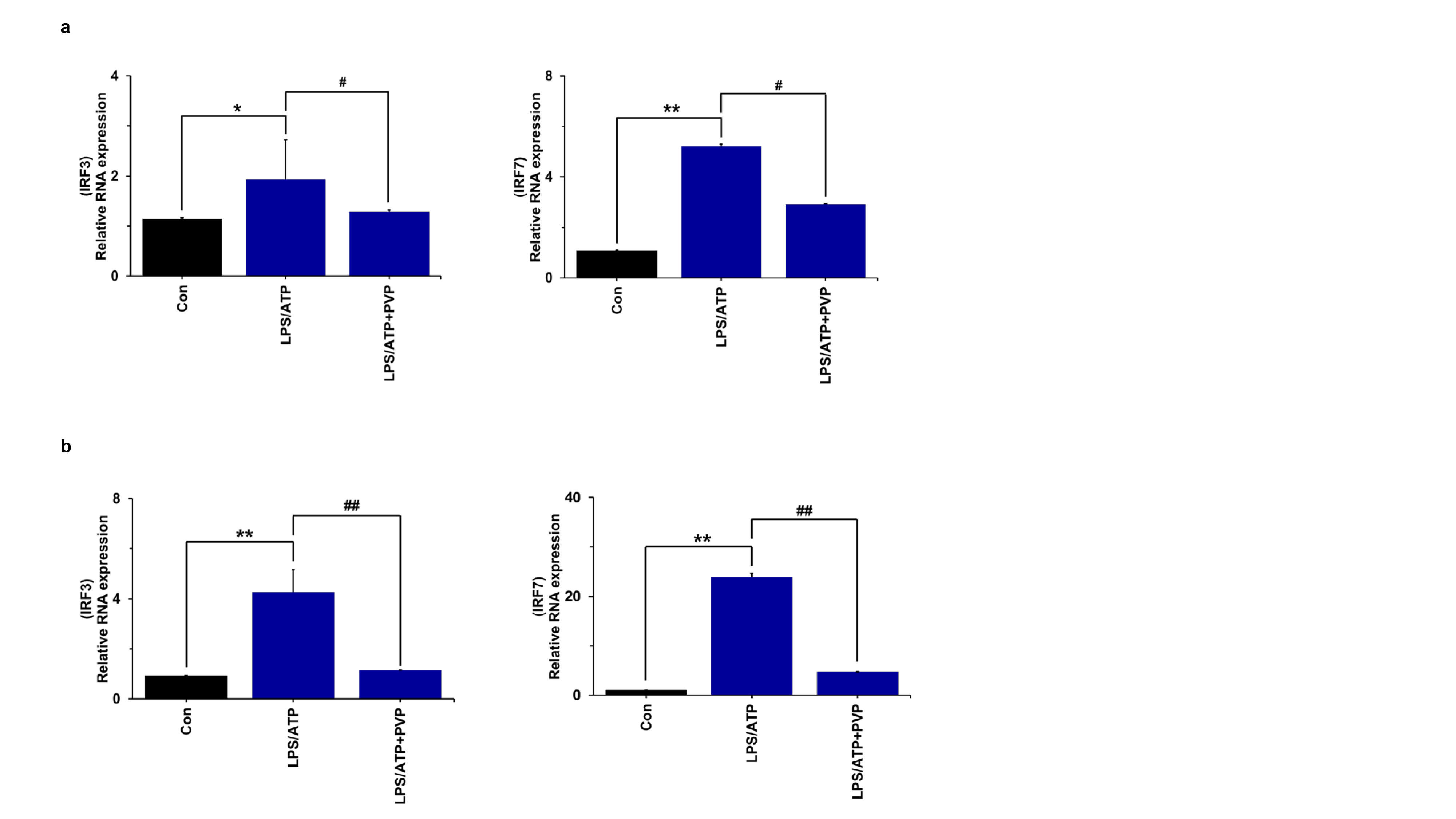

Supplement: Supplementary file 5 — Supplementary Figure 4. [file 41598_2022_7803_MOESM5_ESM.tif]

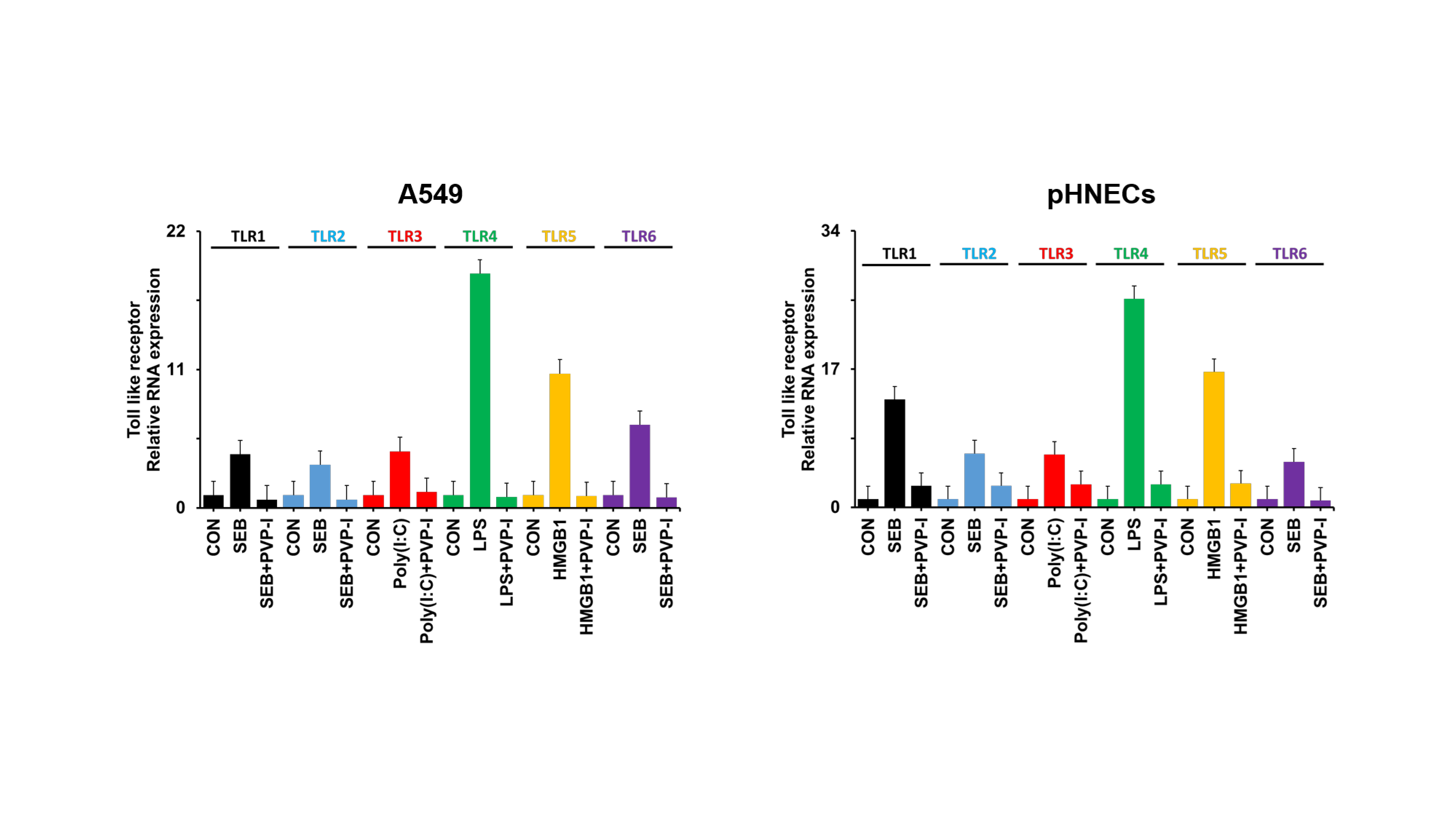

Supplement: Supplementary file 6 — Supplementary Figure 5. [file 41598_2022_7803_MOESM6_ESM.tif]
