# Supplementary material for: Povidone iodine suppresses LPS-induced inflammation by inhibiting TLR4/MyD88 formation in airway epithelial cells
Source: Sci Rep. 2022 Mar 7;12:3681. doi: 10.1038/s41598-022-07803-2 (PMC8901750; doi:10.1038/s41598-022-07803-2)
Supplement: Supplementary file 7 — Supplementary Information. [file 41598_2022_7803_MOESM7_ESM.pdf]

Figure 1a. Original blots

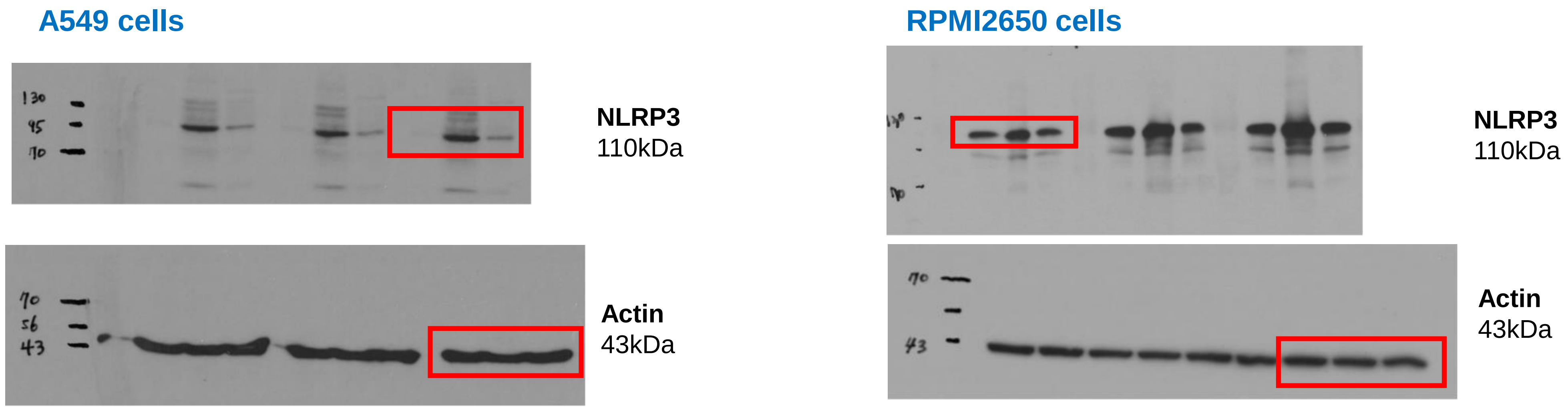

Figure 1c. Original blots

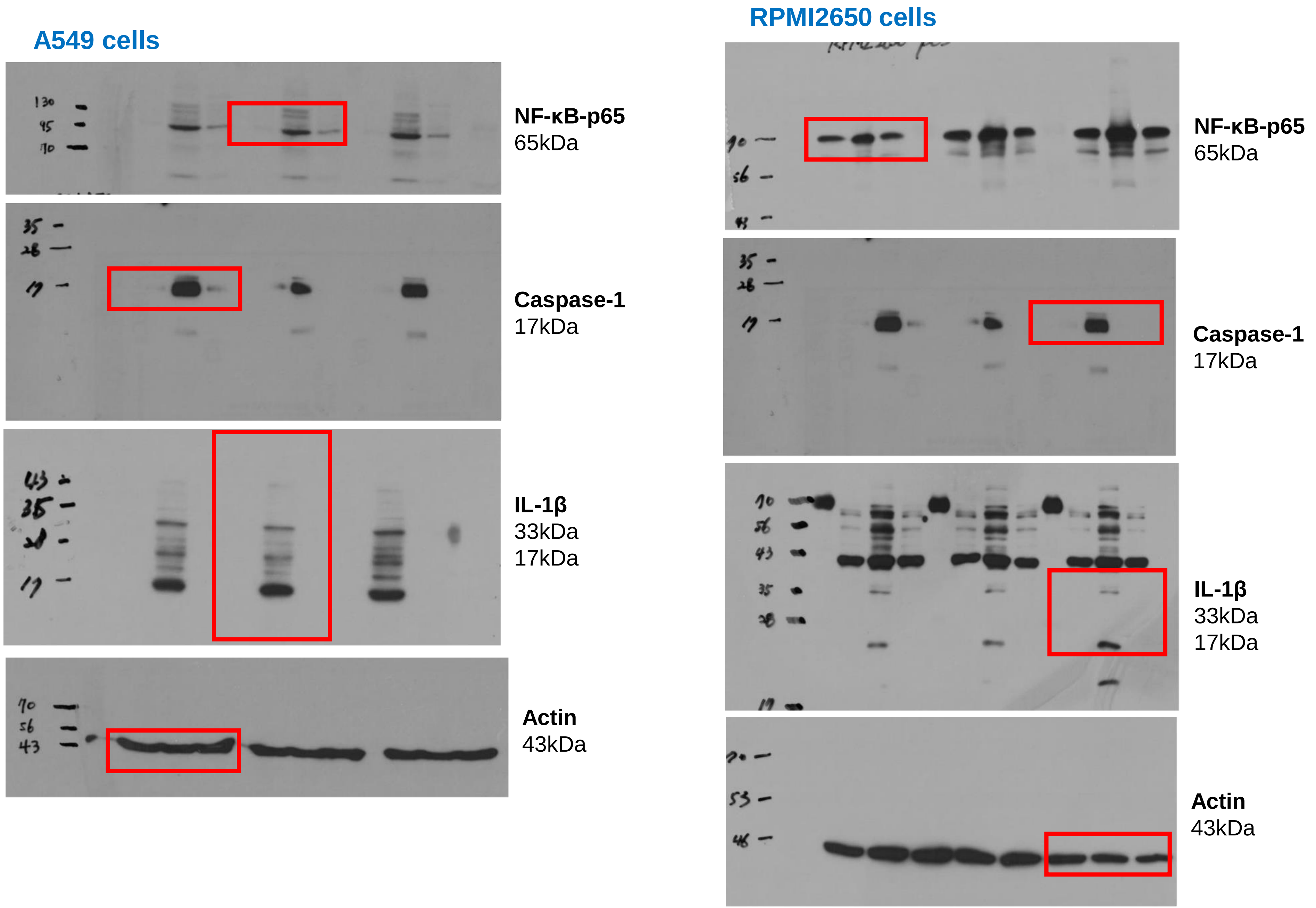

Figure 1e. Original blots

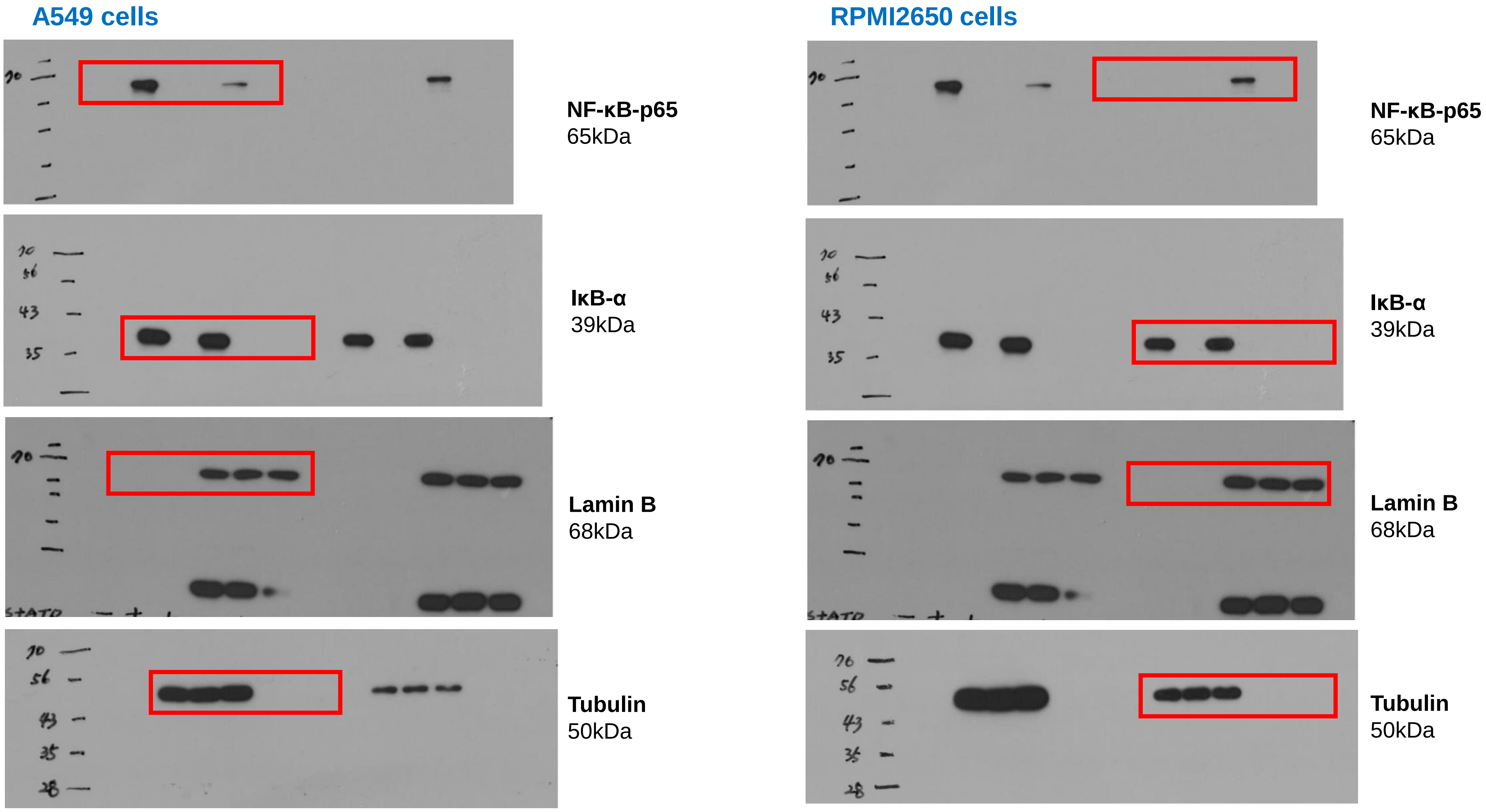

Figure 3c. Original blots

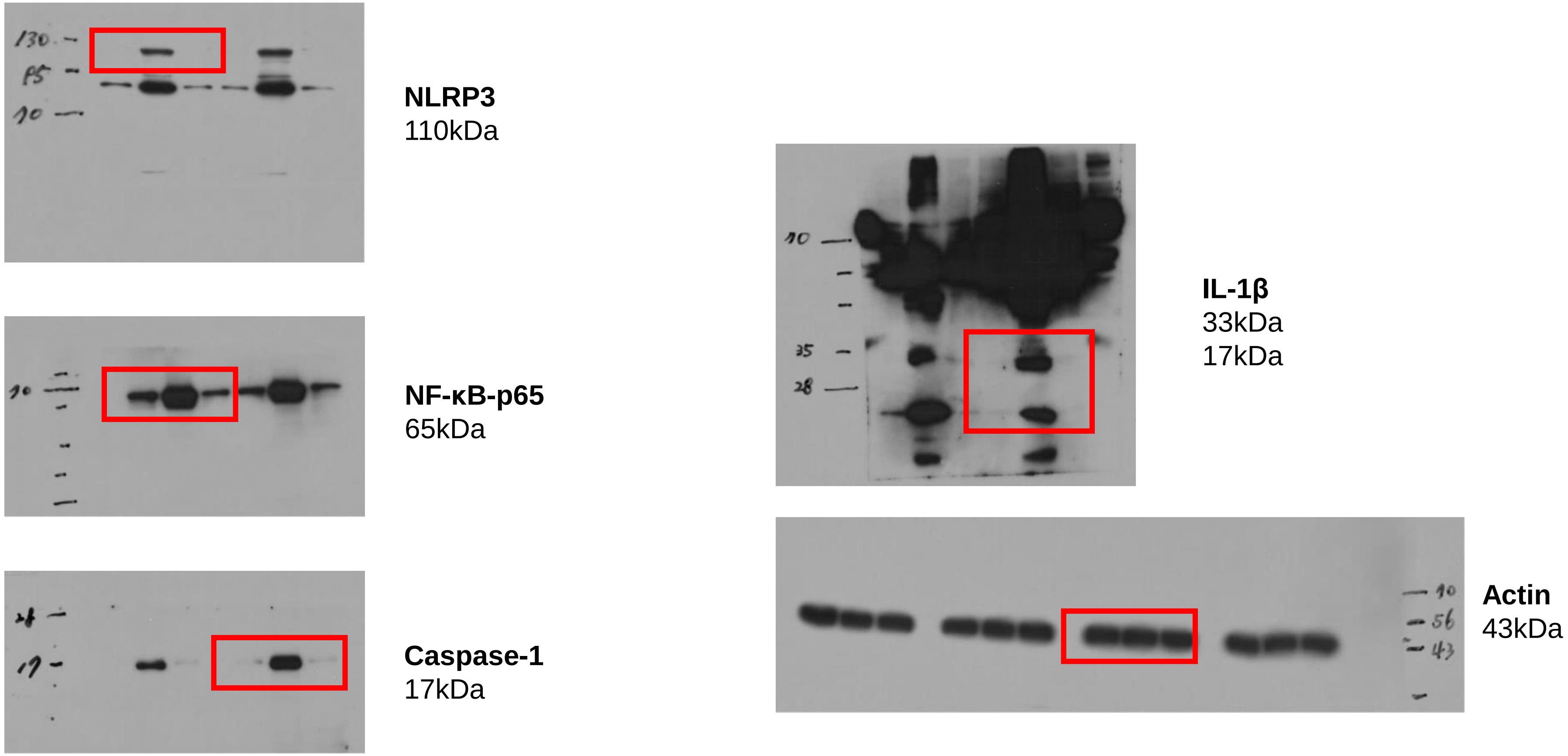

Figure 3d. Original blots

pHNECs

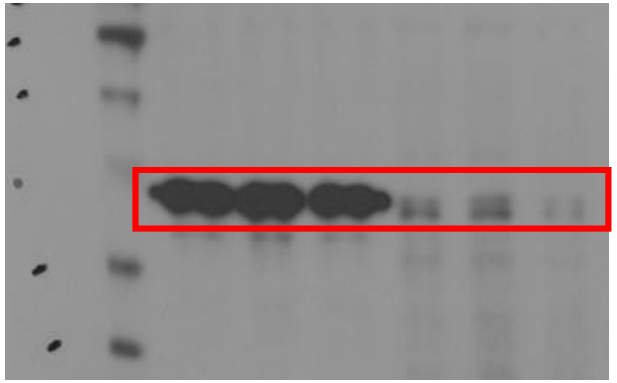

NF-κB-p65
Low exposure time
65kDa

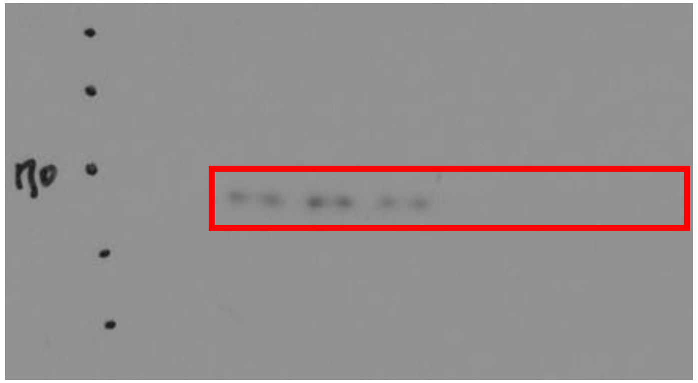

NF-κB-p65
High exposure time
65kDa

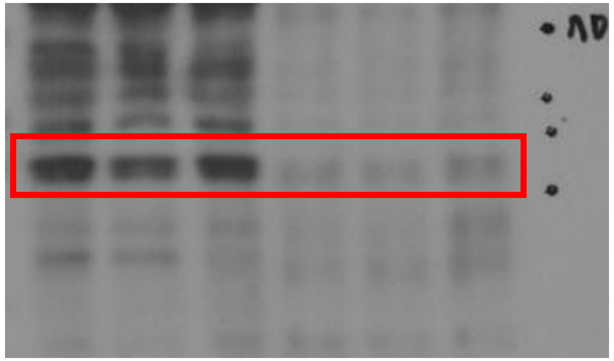

IκB-α
39kDa

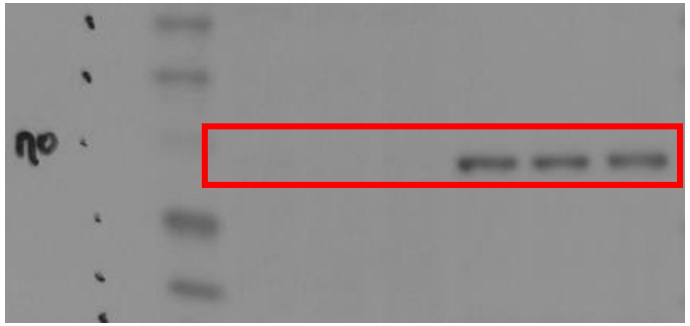

Lamin B
68kDa

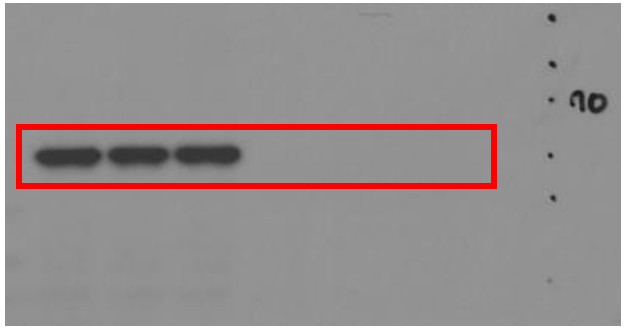

Tubulin
50kDa

Figure 5a. Original blots

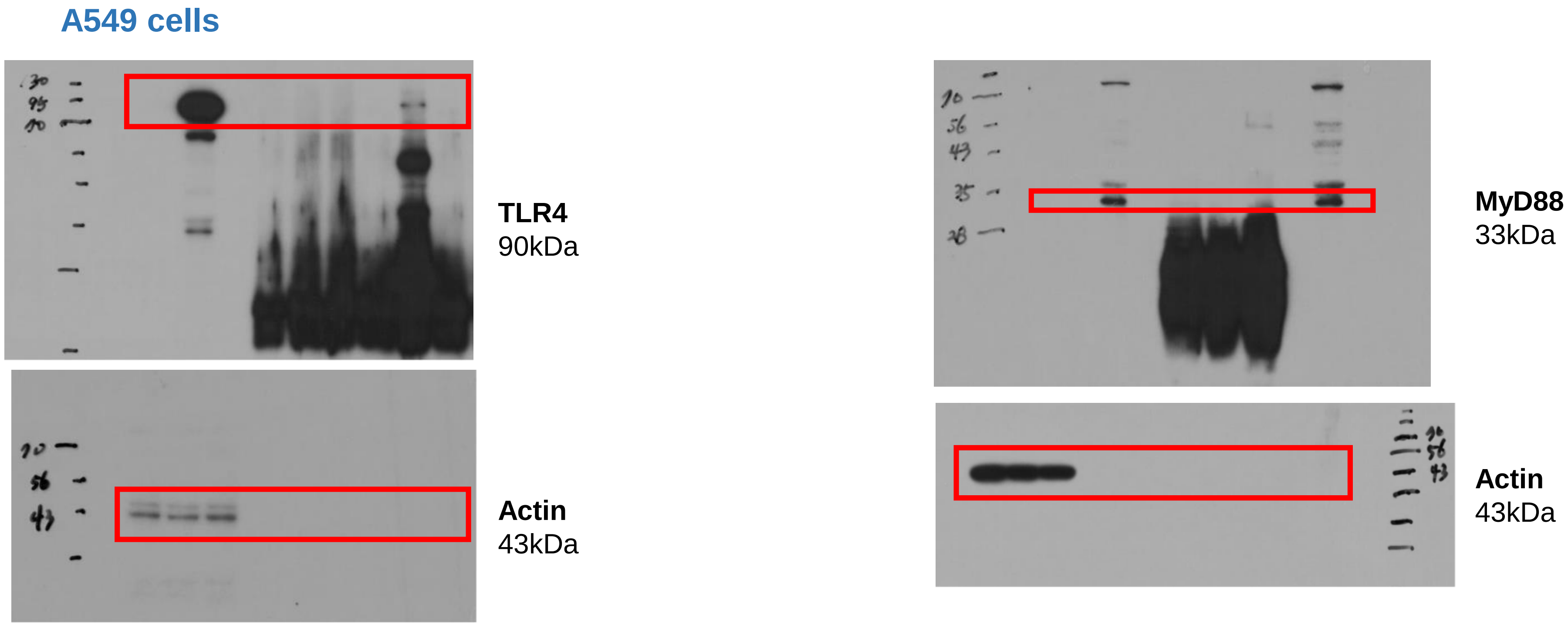

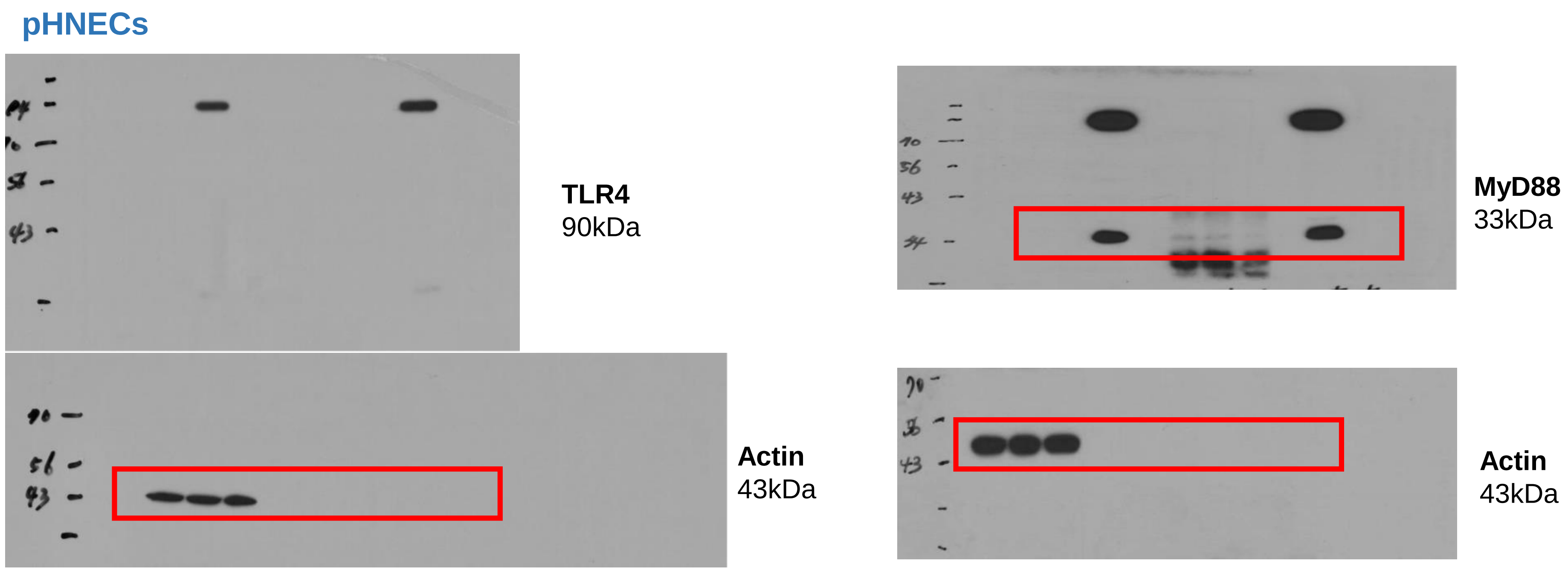
pHNECs
TLR4
90kDa
MyD88
33kDa
Actin
43kDa
Actin
43kDa

Figure 6e. Original blots

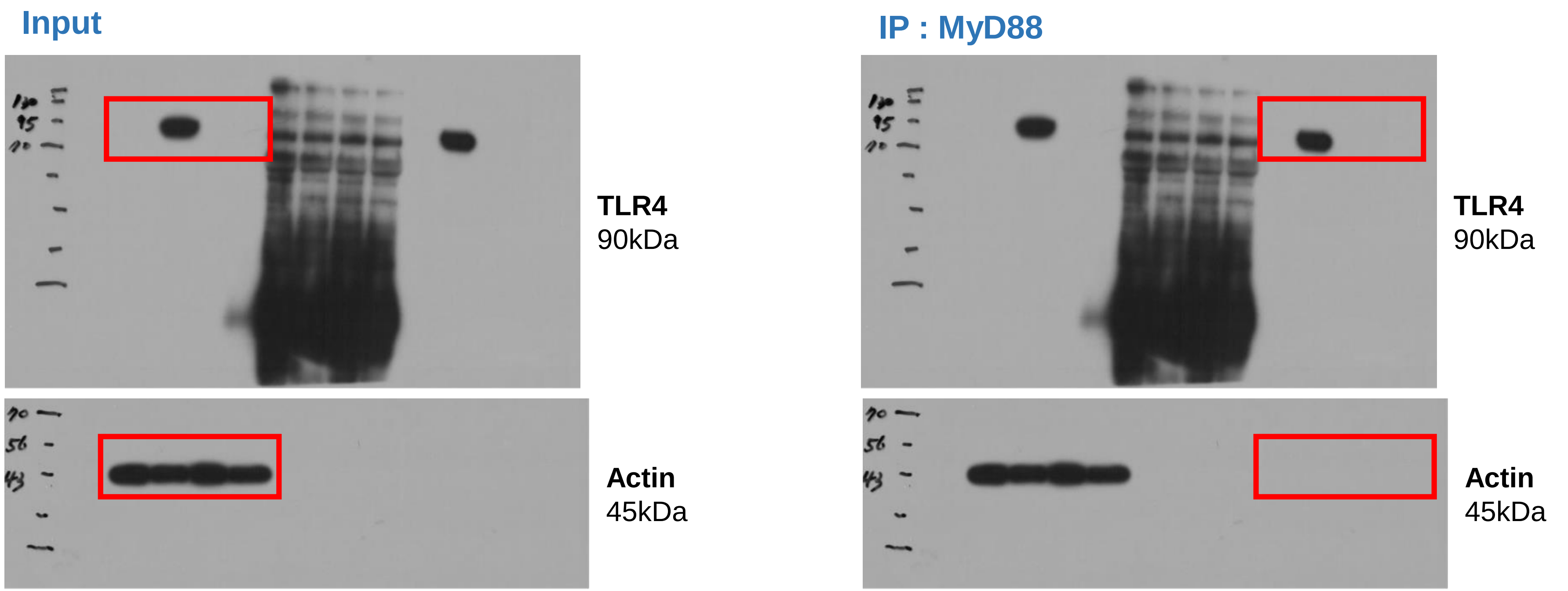

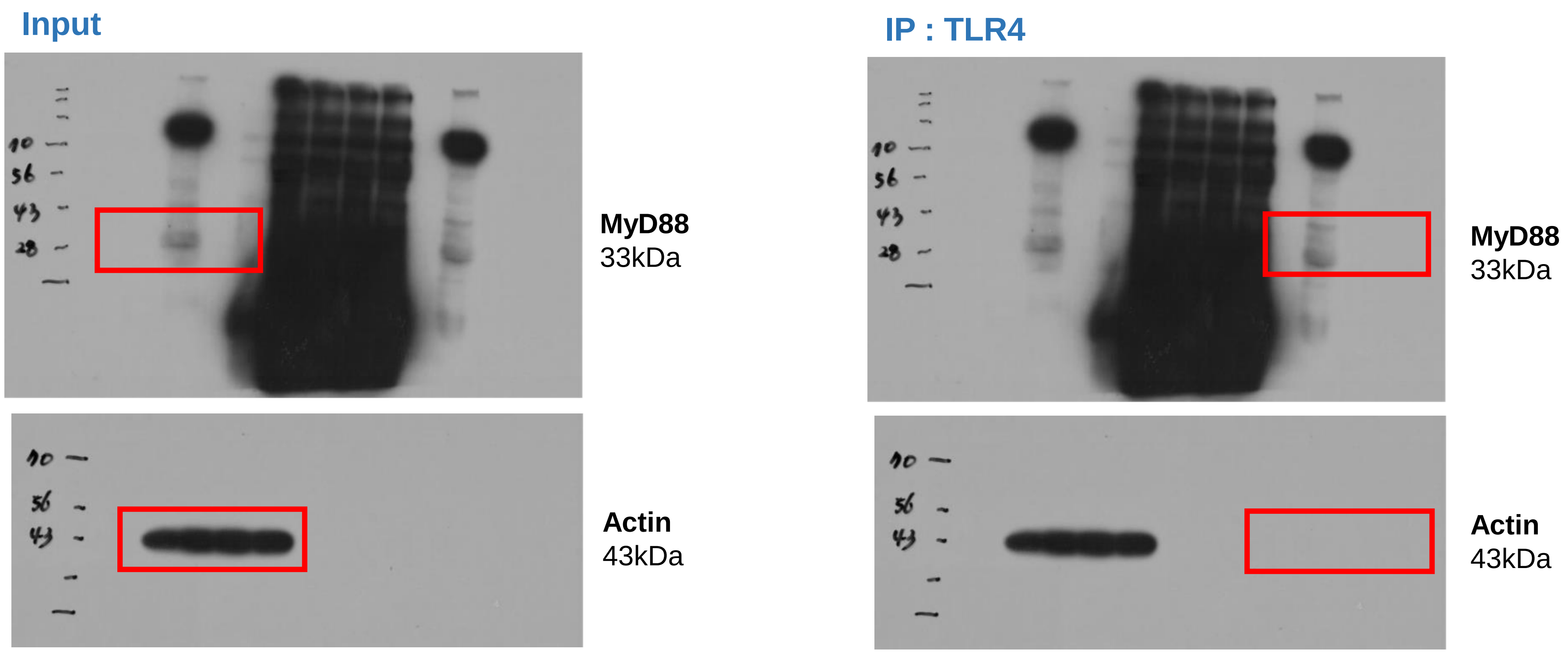
Input
IP : TLR4
MyD88
33kDa
Actin
43kDa
MyD88
33kDa
Actin
43kDa

Figure 7b. Original blots

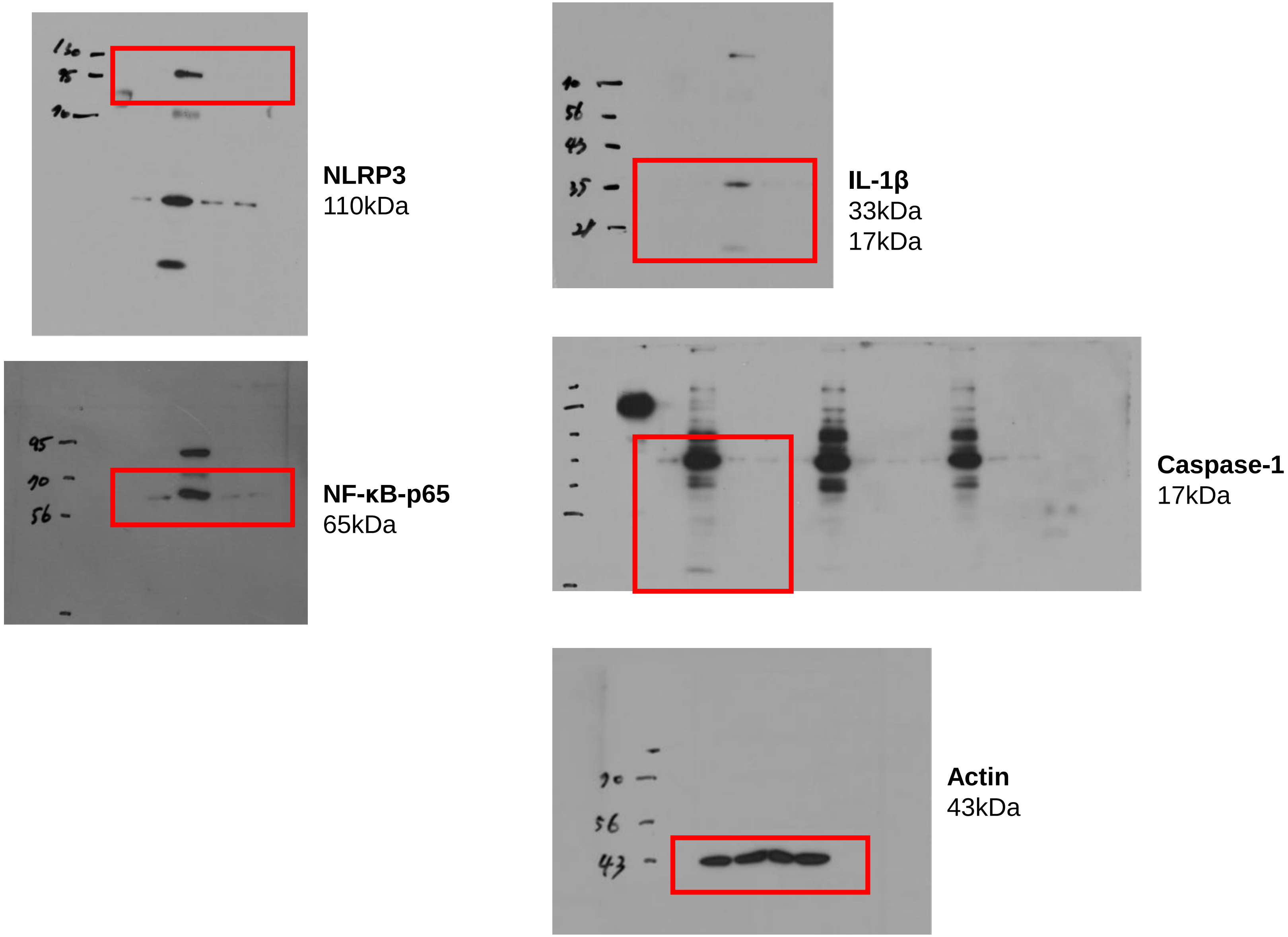

Sup Figure 1b. Original blots

A549 cells

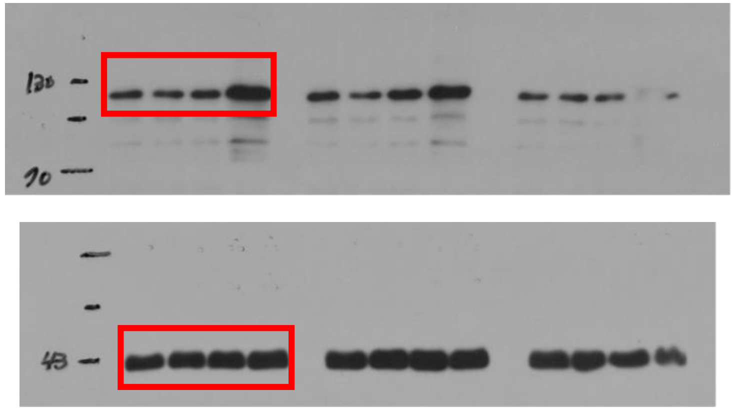

NLRP3
110kDa

Actin
43kDa

RPMI2650cells

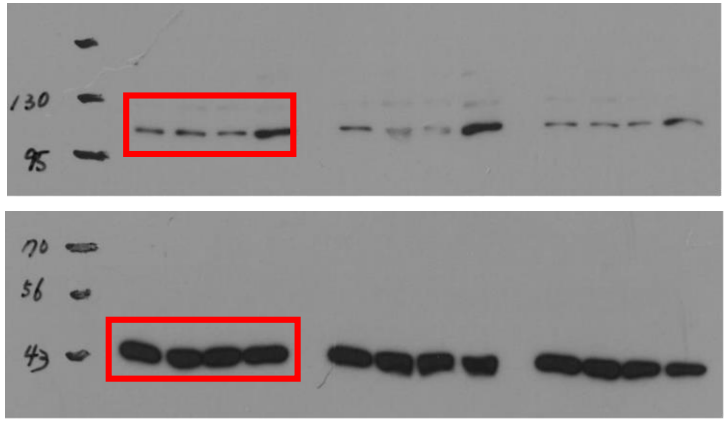

NLRP3
110kDa

Actin
43kDa
